# Structures and Spectroscopic Properties of Polysulfide Radical Anions: A Theoretical Perspective

**DOI:** 10.3390/molecules28155654

**Published:** 2023-07-26

**Authors:** Tristram Chivers, Richard T. Oakley

**Affiliations:** 1 Department of Chemistry, University of Calgary, Calgary, AB T2N 1N4, Canada; 2 Department of Chemistry, University of Waterloo, Waterloo, ON N2L 3G1, Canada

**Keywords:** polysulfide chemistry, radical anions, structures, spectroscopic properties, time-dependent density functional theory

## Abstract

The potential involvement of polysulfide radical anions S*_n_*^•−^ is a recurring theme in discussions of the basic and applied chemistry of elemental sulfur. However, while the spectroscopic features for *n* = 2 and 3 are well-established, information on the structures and optical characteristics of the larger congeners (*n* = 4–8) is sparse. To aid identification of these ephemeral species we have performed PCM-corrected DFT calculations to establish the preferred geometries for S*_n_*^•−^ (*n* = 4–8) in the polar media in which they are typically generated. TD-DFT calculations were then used to determine the number, nature and energies of the electronic excitations possible for these species. Numerical reliability of the approach was tested by comparison of the predicted and experimental excitation energies found for S_2_^•−^ and S_3_^•−^. The low-energy (near-IR) transitions found for the two acyclic isomers of S_4_^•−^ (C_2h_ and C_2v_ symmetry) and for S_5_^•−^ (C_s_ symmetry) can be understood by extension of the simple HMO *π*-only chain model that serves for S_2_^•−^ and S_3_^•−^. By contrast, the excitations predicted for the *quasi*-cyclic structures S*_n_*^•−^ (*n* = 6–8) are better described in terms of *σ* → σ* processes within a localized 2c-3e manifold.

## 1. Introduction

Polysulfide radical anions S*_n_*^•−^ (*n* = 2–8) play a pivotal role as intermediates in the sulfur ↔ sulfide redox cycle [1,2,3,4]. The influence of these short-lived species is frequently invoked in contemporary investigations of sulfur chemistry, including alkali-metal-sulfur batteries [5,6,7], organic syntheses [8], biological chemistry [9,10], geochemical processes involving metal transport [11,12,13] and quantum-dot sensitized solar cells [14,15]. In solution, polysulfide radical anions are readily oxidized by atmospheric oxygen, but the smaller members can be trapped in an aluminosilicate matrix and are known to be the chromophores in yellow (S_2_^•−^), blue (S_3_^•−^) and green (simultaneous presence of S_2_^•−^ and S_3_^•−^) ultramarines [16] and related sodalite-group minerals [17]. The diatomic S_2_^•−^ and the triatomic S_3_^•−^ (C_2v_) radical anions are readily detected in solution or in the solid state by their characteristic UV-visible, Raman or EPR spectra [18]. Indeed, one or more of these techniques is commonly invoked to provide evidence for the role of S_3_^•−^ as an in-situ generated reagent in organic synthesis [8].

In contrast to the well-established spectroscopic signatures of S_2_^•−^ and S_3_^•−^, evidence for the larger members of the family (*n* = 4–8) is fragmentary and often conflicting. Of these species, S_4_^•−^ has a long but somewhat checkered history. In 1970, as part of his pioneering study on solutions of alkali-metal polysulfides in electron-pair donor solvents, e.g., DMF, HMPA, Seel attributed a visible absorption at ca. 515 nm to S_4_^•−^ [19], but later the band was reassigned to a dimer [20], an example of which has recently been structurally characterized in a dinuclear Bi(III)Bi(III) complex [21]. In 1983 Clark et al. investigated the nature of the sulfur chromophore in ultramarine pink by Raman spectroscopy [22], but they were unable to distinguish between S_4_ and S_4_^•−^ (or even S_3_Cl). The association of a 490 nm band with S_4_^•−^ has nonetheless persisted [9,23], and Chiba and co-workers have recently invoked formation of the radical anion during the photolysis of the closed-shell dianion S_4_^2−^ [24,25,26,27]. The use of other techniques to identify S_4_^•−^ in solution, notably Raman and IR spectroscopy [28,29,30], has been pursued, but band assignments based on calculated vibrational frequencies have been questioned [3]. Likewise, an EPR signal observed in solutions of lithium polysulfide solutions in DMF was proposed to belong to S_4_^•−^ [31], but the isotropic *g*-value (2.031) lies close to that of the dominant radical anion S_3_^•−^ (2.029) [32]. In principle, high field EPR spectroscopy could be used to distinguish between these (and other) polysulfide radical anions, and on this basis Chukanov et al. recently suggested the presence of S_4_^•−^ in various sodalite minerals [33].

**Scheme 1:**
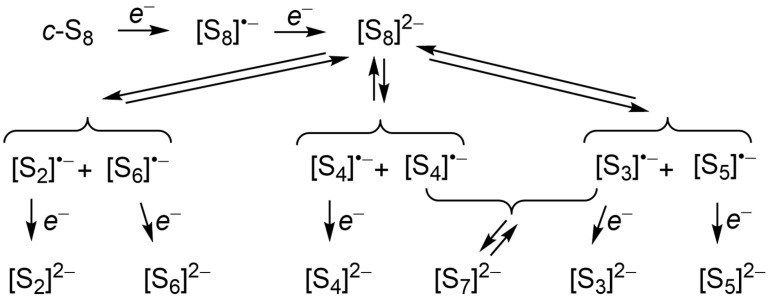
Possible radical anion intermediates in the stepwise electrochemical reduction of *c*-S_8_.

The larger anions S*_n_*^•−^ (*n* = 5–8) are also acknowledged as potentially important intermediates in the S_8_ ↔ S^2−^ redox processes, as in the electrochemical reduction of cyclo-S_8_, redox transformations in alkali metal-sulfur batteries [27] and the formation of polysulfides from photoexcited quantum dots [14,15]. Exploration of the stepwise electrochemical reduction of cyclo-S_8_ in non-aqueous solvents has been extensively pursued, with formation of S_8_^2−^ generally accepted by the battery community to occur first (Scheme 1) [3]. Initially, in 1970, Merritt and Sawyer claimed the preliminary formation of the one-electron reduction product S_8_^•−^ [34], then revised this interpretation to a two-electron transfer [35], in agreement with the work of Bonnaterre and Cauquis [36] and also supported by results obtained by Hardacre and coworkers using ionic liquids as the solvent medium [37]. However, in 2008, the results of a detailed cyclic voltammetric study of the reduction of S_8_ in various solvents were consistent with the formation of S_8_^2−^ via two consecutive one-electron steps [38]. The potential for the involvement of S_4_^•−^ in the S_8_ reduction process has been argued [39,40,41,42], but in the absence of a clear spectroscopic signature for the anion, the issue has not been resolved. Although symmetrical dissociation of S_8_^2−^ to give two S_4_^•−^ radical anions is calculated to be exergonic [43], and an absorption band at ca. 700 nm was assigned to S_4_^•−^ [44,45] in spectrochemical studies of the reduction of sulfur in DMSO and DMF, others insist that it has never been detected [46].

Surprisingly, although the preparation and structural characterization of several salts of the S_8_^2−^ were reported more than 30 years ago [47,48], there is limited information on the behavior of ion-separated salts of S_8_^2−^ in non-aqueous solvents. Formation of S_8_^2−^ from S_8_ has traditionally been interpreted to be followed by disproportionation to S_6_^2−^ and ¼S_8_ [39,43,49], the former dissociating to afford S_3_^•−^ [48]. However, a disproportionation process simply represents a mass balance, and belies the reality that the formation of an eight-membered S_8_ ring must involve the intermediacy of long chain polysulfide dianions S*_n_*^2−^ with *n* > 8 and, possibly, polysulfide radical anions such as S*_n_*^•−^ (*n* = 4, 6) [3]. Alternative fates for S_8_^2−^ in dilute solution can be envisaged (Scheme 1) in terms of equilibria involving its symmetric and asymmetric dissociation to afford, in principle, the entire series of polysulfide radical anions S*_n_*^•−^ (*n* = 2–6). Longer chain closed-shell dianions such as S_10_^2−^ and S_12_^2−^, salts of which have recently been characterized [50,51], can then be viewed as arising from the reverse process, that is, symmetric coupling of the radical anions S_5_^•−^ and S_6_^•−^, respectively, while association of S_3_^•−^ and S_4_^•−^ yields S_7_^2−^. Unfortunately, information on the electrochemical reduction of the cyclic allotropes S_6_ and S_7_ is lacking. That being said, in 2002 Dehnicke and coworkers isolated and structurally characterized crystals of the (orange/red) radical ion salt [Ph_4_P][S_6_], the only such characterization of an ion-separated salt of a polysulfide radical anion [52]. The importance of this result is discussed below.

As demonstrated in this brief survey, there are many unsettled questions regarding the basic chemistry of elemental sulfur, in particular relating to the stability, structure and properties of radical ion products that may be generated during the sequential reduction of cyclo-S_8_ or the oxidation of the sulfide ion S^2−^ [1]. These questions have given rise to ongoing controversies, many relating to the colors of these ephemeral species—what do they look like, and do they exist if they cannot be seen?

While the colors of S*_2_*^•−^ and S*_3_*^•−^ are well characterized, the optical properties of the larger *putative* polysulfide radical anions have been explored only to a very limited extent. Fabian et al. used density functional theory (DFT) methods to probe the excited states of S_4_^•−^, and predicted a strong absorption in the near-IR region, with a weaker band near 350 nm for cis S_4_^•−^(C_2v_) isomer, which is slightly more stable than the trans (C_2h_) isomer [53]. More recently, and using DFT and CASSCF methods, Rejmak confirmed that the cis S_4_^•−^ radical anion could be identified by a strong absorption in the near-IR region [54] and proposed that the red chromophore in ultramarine red is *neutral* S_4_ rather than the corresponding radical anion. Surprisingly, the excited state properties of the remaining radical ions in the series, that is S*_n_*^•−^ (*n* = 5–8), have never been explored theoretically, perhaps because even their ground state geometries have remained somewhat of a puzzle.

The principal objective of the present article is to redress this issue, to fill in the blanks not only in regard to the spectroscopic signatures of these radical anions, that is, their excited state properties, but also to establish their ground state structures, particularly in solution in polar solvents, the media in which they are most likely to be generated.

## 2. Results

### 2.1. Structural Trends

In the following sections we describe the structural features and relative energies provided by spin unrestricted PBE0/D3/def2-QZVP calculations for the family of radical anions S*_n_*^•−^ (*n* = 4–8). The results build upon the earlier systematic studies of Hunsicker et al. [55], Steudel [40] and Wong [56], but include several alternative shapes not previously considered. The possible effects of solvation are heavily stressed, as our overall aim has been to identify structures most likely to be present in solution in the polar solvents typically used for the spectroscopic observation of these species. To this end we performed not only standard “gas phase” geometry optimizations but also optimizations employing the polarized continuum model (PCM) to simulate solvation effects, with DMF (*ε* = 37.2) serving as a representative example. As observed by Steudel, the inclusion of solvation using the PCM approach leads to only minor geometrical adjustments, and for this reason only the “gas phase” structural parameters are presented in the main text (see Figure S1 for PCM-adjusted numbers). Solvation effects, however, have important energetic consequences, favoring structures with large molecular dipoles, and can play a pivotal role in adjusting the balance between structural alternatives which are otherwise closely matched energetically.

#### 2.1.1. S_2_^•−^ and S_3_^•−^

Like molecular oxygen, the diatomic molecule S_2_ possesses a triplet ground state [57]. Addition of an electron to one of the two half-filled *π*_g_* orbitals, to afford the ^2^Π_g_ radical anion S_2_^•−^, leads to an elongation of the S–S bond, calculated here = 1.996 Å. Attachment of a third sulfur introduces the possibility of structural options for S_3_^•−^, namely linear (D_∞h_), equilateral and isosceles triangular (D_3h_ and C_2v_, respectively); the last is established as the energetically preferred. Structural parameters calculated here for the ^2^B_1_ state, an S–S bond distance of 1.984 Å and inter-bond angle of 115.7°, are consistent with previous estimates [58,59].

#### 2.1.2. S_4_^•−^

**Figure 1:**
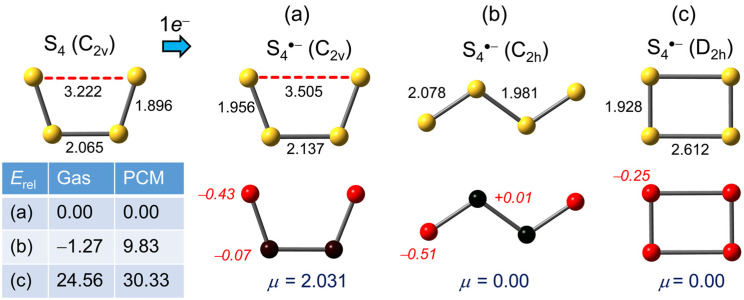
(U)PBE0/D3/def2-QZVP optimized geometries (distances in Å) for S_4_^•−^, with Mulliken charges (in italics) and dipole moments *μ* (in Debye). Relative gas phase and PCM (=DMF) corrected total energies *E*_rel_ are in kJ mol^−1^.

Based on both experimental and theoretical evidence [48,49,60] the geometry of a discrete neutral S_4_ molecule displays C_2v_ symmetry, consisting of a planar broken-ring structure with one “long” S–S bond, calculated here = 3.222 Å. The most appealing option for the corresponding S_4_^•−^anion is also a planar C_2v_ structure (Figure 1a), akin to the neutral form but with the “long” S–S bond further stretched (calculated here = 3.505 Å). However, a C_2h_ isomer (Figure 1b), generated from the C_2v_ by a 180° rotation about the central S–S linkage, is also possible.

Frequency calculations confirm that both the C_2v_ (cis) and C_2h_ (trans) forms are true energetic minima, the latter being slightly more stable in the gas phase. But the energetic competition between the two ceases upon inclusion of PCM (=DMF) solvation, with the centrosymmetric (*μ* = 0) C_2h_ isomer rising relative to the C_2v_ form by nearly 10 kJ mol^−1^. The presence of both isomers in solvents with a low dielectric constant may nonetheless be possible. Other structures, based on closed rings, have been explored by previous workers and found to be energetically much more high-lying. Re-examination here of these variants, none of which represents a true energetic minimum, indicates the centrosymmetric (*μ* = 0) D_2h_ modification (Figure 1c), formed by a Jahn–Teller distortion of a putative D_4h_ geometry [61], is the most stable of the closed-ring group, although it still lies well above the C_2v_ form and, with the inclusion of PCM, its relative energy rises even higher.

#### 2.1.3. S_5_^•−^

**Figure 2:**
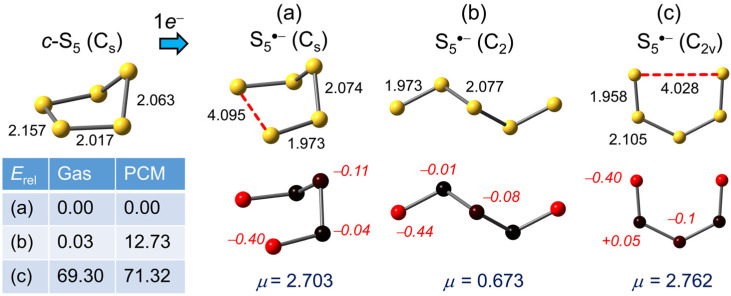
(U)PBE0/D3/def2-QZVP optimized geometries (distances in Å) for S_5_^•−^, with Mulliken charges (in italics) and dipole moments *μ* (in Debye). Relative gas phase and PCM (=DMF) corrected total energies *E*_rel_ are in kJ mol^−1^.

While the structure of neutral S_5_ is unknown, an open envelope-like or chair shape with C_s_ symmetry has been predicted in previous studies [62,63], with the S–S bond bisected by the mirror plane slightly elongated. We concur with this result, and calculate the unique S–S distance = 2.157 Å. The apparent weakening may be attributed, in valence bond parlance, to lone-pair repulsion arising from the eclipsed alignment of the two neighboring S–S bonds. One-electron reduction to the radical anion S_5_^•−^ leads to a variety of structural alternatives, the most obvious involving complete separation (to 4.095 Å) of the already weakened mirror-bisected linkage, to afford the distorted C_s_ chair illustrated in Figure 2a. Vibrational analysis confirms that the optimized structure represents a true energetic minimum and, as indicated by the associated Mulliken charge densities, negative charge is heavily localized on the two sulfurs associated with the “broken” bond. As expected, charge polarization, and its impact on the molecular dipole, increases with the inclusion of PCM (Table S1).

Open-chain structures for S_5_^•−^ are also possible; several variants have been explored by previous workers, but in our hands these all gravitate on optimization towards the twisted chain (C_2_ symmetry) minimum shown in Figure 2b. It is almost co-energetic with the C_s_ chair, perhaps not surprisingly as the two structures are interconvertible by a 180° rotation of one of the terminal bonds. However, by virtue of the lower dipole moment of the open-chain form, a substantial gap opens with the inclusion of PCM. In addition, we have considered two “forced” planar modifications, one being the cis-cis C_2v_ geometry shown in Figure 2c. While it does not represent a stable minimum, and its relative energy is substantially higher than the related C_s_ chair, its electronic structure provides a useful conceptual link (vide infra) to the shorter chain anions (*n* = 2–4). For the corresponding cis-trans isomer, which is isostructural with the closed shell SSNSS^−^ anion [63,64], the energy gap is considerably less, both in the gas phase (26.1 kJ mol^−1^) and in DMF (32.6 kJ mol^−1^), but is still not a true minimum.

#### 2.1.4. S_6_^•−^

**Figure 3:**
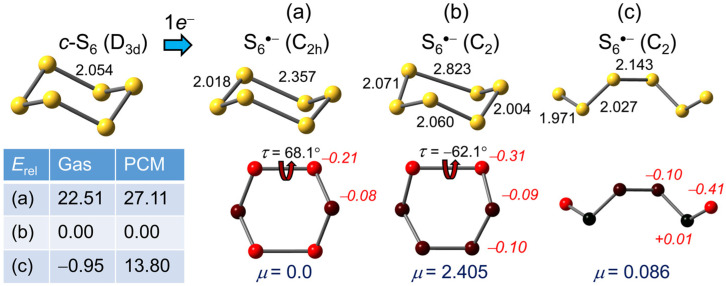
(U)PBE0/D3/def2-QZVP optimized geometries (distances in Å) for S_6_^•−^, with Mulliken charges (in italics) and dipole moments (*μ*) in Debye. Relative gas phase and PCM (=DMF) corrected total energies *E*_rel_ are in kJ mol^−1^.

Here we have the unique advantage of experimental structural information on both the neutral molecule and its radical anion. The cyclic, chair-shaped structure of S_6_, with D_3d_ symmetry and all neighboring bonds staggered, has been characterized crystallographically [65]; the observed S–S distance = 2.057(18) Å compares well with the value calculated here = 2.054 Å (Figure 3). In the corresponding radical anion S_6_^•−^, identified in the crystal structure of the tetraphenylphosphonium salt [Ph_4_P][S_6_], the cyclic chair shape is retained (Figure 3a), despite some disorder, but with two elongated S–S bonds = 2.634(4) Å [47]. In their report, however, the authors cautioned that the apparently high molecular symmetry (C_2h_) observed for the anion might be dictated by the high lattice symmetry (space group *C*2/*c*), and provided BP86/TZ2P results indicating that a distorted chair structure (Figure 3b) with C_2_ symmetry was actually more stable.

From a theoretical perspective, one-electron reduction of the high-symmetry geometry of neutral S_6_ gives rise to an orbitally degenerate ground state for the resulting radical anion S_6_^•−^. Thus, when using D_3h_ symmetry constraints as a starting point for a geometry optimization, the symmetric chair immediately breaks symmetry and undergoes a first-order Jahn–Teller distortion [61] to C_2h_ symmetry, affording two elongated S–S bonds, calculated here = 2.357 Å (Figure 3a), somewhat shorter than that observed experimentally. However, as observed earlier, while this centrosymmetric C_2h_ structure represents a stationary point it is not an energy minimum. Upon release of symmetry constraints, it undergoes a second-order distortion to the C_2_ modification (Figure 3b), in which one of the two elongated S–S bonds in the C_2h_ geometry stretches further to 2.823 Å, a result in accord with the earlier DFT work [47]. By our calculations the energy difference between the C_2h_ and C_2_ structures is large (22.5 kJ mol^−1^), even in the gas phase, and increases to 27.5 kJ mol^−1^ with the inclusion of PCM (*μ* = 0 in the C_2h_ form).

In addition to the nominally closed-ring variants for S_6_^•−^ several open chain options have been considered. Of these, we find the lowest energy C_2_ structure (Figure 3c), which can be converted into the *quasi*-cyclic form by a ca. 180° rotation about the central S–S bond, constitutes a true minimum. Predictably, in the gas phase the total energies of the two rotamers are almost identical, but in accord with the low dipole moment of the open chain form the balance changes sharply in favor of the ring structure when the PCM is included.

#### 2.1.5. S_7_^•−^

**Figure 4:**
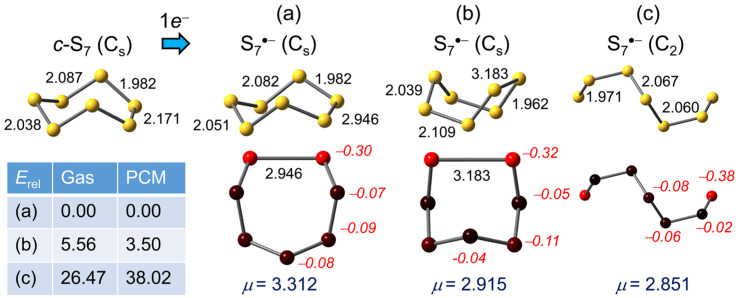
(U)PBE0/D3/def2-QZVP optimized geometries (distances in Å) for S_7_^•−^, with Mulliken charges (in italics) and dipole moments (*μ*) in Debye. Relative gas phase and PCM (=DMF) corrected total energies *E*_rel_ are in kJ mol^−1^.

Neutral S_7_ possesses a chair-like structure with C_s_ symmetry [66,67], with the unique mirror-bisected bond lengthened to 2.18 Å (calculated here = 2.171 Å) by the effects of lone-pair repulsion occasioned by the eclipsed alignment of the neighboring bonds, as seen in *c*-S_5_. In the structure of the global energetic minimum for S_7_^•−^ the cyclic chair motif found in the neutral molecule is retained, but the already weakened mirror-bisected linkage is lengthened to 2.946 Å in the radical anion (Figure 4a), with the associated Mulliken charge densities heavily localized on the two sulfurs linked by the weakened bond.

Not surprisingly, a boat-shaped conformation (Figure 4b), in which the unique S–S bond is a little longer (3.183 Å) than in the chair, is also possible. This feature may be of relevance to optical properties, as its relative energy lies only slightly above that of the chair in both the gas phase and solution, so that the two conformers may coexist in equilibrium in solution. Outside of this pair of *quasi*-cyclic structures there is an open chain variant with C_2_ symmetry (Figure 4c). It represents a local energy minimum, but is significantly less stable than the chair/boat structures in the gas phase, the gap increasing when PCM is invoked.

#### 2.1.6. S_8_^•−^

**Figure 5:**
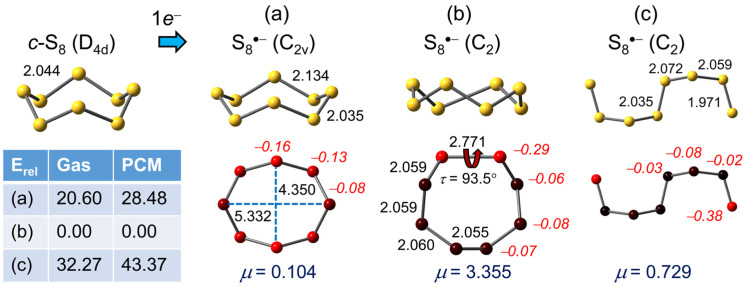
(U)PBE0/D3/def2-QZVP optimized geometries (distances in Å) for S_8_^•−^, with Mulliken charges (in italics) and dipole moments (*μ*) in Debye. Relative gas phase and PCM (=DMF) corrected total energies *E*_rel_ are in kJ mol^−1^.

The eight-membered ring found in orthorhombic *α*-sulfur displays a classic crown conformation with D_4d_ symmetry, with all neighboring bonds (measured at 2.055(2) Å, calculated here = 2.044 Å) mutually staggered [68]. The structural changes accompanying formation of S_8_^•−^ follow a similar pattern to that seen for S_6_^•−^. Addition of an electron to the cyclo-S_8_ in D_4d_ symmetry affords a degenerate ground state for the resulting radical anion, thereby setting up a first order Jahn–Teller distortion [61], which in this case affords the “squeezed” C_2v_ crown geometry shown in Figure 5a.

While this high-symmetry structure is not an energy minimum, the possibility of trapping it in a crystal lattice, as in the case of the C_2h_ form of S_6_^•−^, is worthy of consideration. That being said, upon release of all symmetry constraints the C_2v_ structure evolves into a C_2_ variant (Figure 5b) which, like the C_2_ structure of S_6_^•−^, displays one elongated S–S bond, calculated here = 2.771 Å. Outside of distorted crown geometries, there are few energetically viable alternatives. Of these, the open-chain C_2_-symmetry motif (Figure 5c) represents the only true minimum, but its energy lies well above that of the C_2_ crown. Given its relatively low polarity, inclusion of PCM further widens the energy gap.

In summary, the smaller members (*n* = 3, 4) of the polysulfide radical anion family adopt open chain structures, in part because they have no choice, as there is too much ring strain in the cyclic alternatives. That being said, when alternatives exist, as in the C_2v_ (cis) and C_2h_ (trans) options for S_4_^•−^, solvent effects may well dictate the outcome, with the non-centric cis isomer being preferred in polar solvents and the centric trans isomer possibly being viable in non-polar solvents. For medium-sized rings, i.e., *n* = 5, 6, closed or broken-ring structures compete with open-chain variants, and again the choice may depend upon the polarity of the solvent employed, with polar environments or lattice constraints (for *n* = 6)) favoring the cyclic or *quasi*-cyclic modifications. In the following sections we focus on the structures most likely preferred in the latter environments. Finally, when *n* = 7 and 8, the stability of the cyclic structures clearly outranks the open-chain alternatives, regardless of solvent effects. Dynamic equilibria between cyclic and acyclic forms are unlikely, a conclusion which may have consequences for the mechanism of formation of S_8_^2−^ [39].

### 2.2. Electronic Spectra

Using the polar-medium preferred geometries afforded by the unrestricted DFT calculations described above, single point TD-DFT calculations were performed on the radical anions S*_n_*^•−^ (*n* = 2–8), to explore the number, nature and energies of the possible electronic excitations. A compilation of the relevant states, dominant orbital transitions, frequencies *ν*, wavelengths λ and oscillator strengths *f* is provided in Table 1.

As when dealing with geometrical trends, presentation and discussion of the results is developed according to the value of *n*, beginning with the three short-chain anions (*n* = 2–4), where the electronic excitations are all clearly *π* → π*. From there on (*n* = 5–8) the non-planar, distorted or broken-ring geometries militate against the use of conventional *σ*/*π* symmetry descriptors which usually aid with band assignments, but for *n* = 5 the calculated spectrum can still be rationalized by extension of the simple *π*-only model. Finally, the single elongated S–S linkages found in the *quasi*-cyclic structures (*n* = 6–8), which are broadly consistent with localized two-center three-electron (2c-3e) bonds, reminiscent of those found in transient organic disulfide radical anions (RS-SR)^•−^ [69,70], give rise to low energy excitations that are best described as *σ* → σ* processes within the 2c-3e manifold.

#### 2.2.1. S_n_^•−^ (*n* = 2–4)

**Figure 6:**
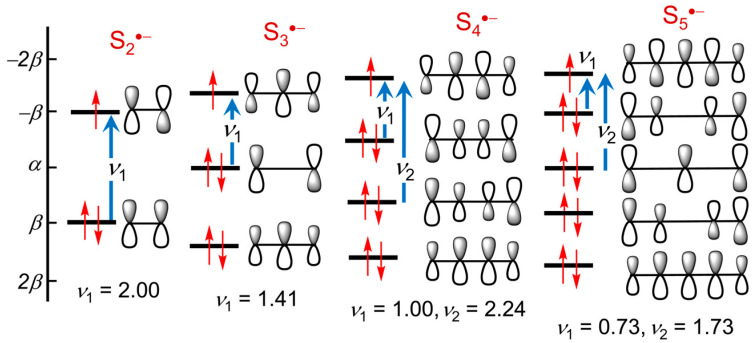
HMO energy levels for arrays of 3*p*-orbitals in model open chain S*_n_*^•−^ (*n* = 2–5) radical anions; excitation energies *ν*_1_, ν_2_ are in units of *β*.

The origin of the electronic excitations in the short-chain radical anions S*_n_*^•−^ (*n* = 2–4) can be readily understood with reference to the manifold of *π*-orbitals predicted by the classical Hückel molecular orbital (HMO) linear chain model [71,72], using linear arrays of overlapping sulfur 3*p*-orbitals as a basis set. For such systems the eigenvalues *e*_j_ are given by the analytical expression *e*_j_ = *α* + 2*β* cos (*jπ*/*N* + 1), where *α* and *β* are the respective Coulomb and resonance parameters, and *N* is the number of orbitals (atomic centers) in the chain. Schematic plots of the resulting *π*-energy levels and MOs are illustrated in Figure 6. Within this framework, a single *π* → π* excitation *ν*_1_ is expected for the diatomic anion S_2_^•−^, with a slightly lower energy *nπ* → π* transition *ν*_1_ anticipated for the triatomic chain S_3_^•−^. Extrapolation to planar open chain S_4_^•−^ systems suggests two excitations *ν*_1_ and *ν*_2_ are possible. Of these, *ν*_1_ is predicted to occur at still lower energy, and its magnitude can be estimated by calibration against the known value of *ν*_1_ for S*_3_*^•−^ (*λ*_max_ = 615–620 nm in DMF or HMPA) [73]. Based on this simple model the first transition ν_1_ in both isomers of S_4_^•−^is predicted to shift well beyond the visible region. For the cis (C_2v_) isomer a second excitation *ν*_2_ is anticipated towards the UV region, while for the trans (C_2h_) form *ν*_2_ should not be observed at all, as it is symmetry-forbidden (*g* → *g*).

**Figure 7:**
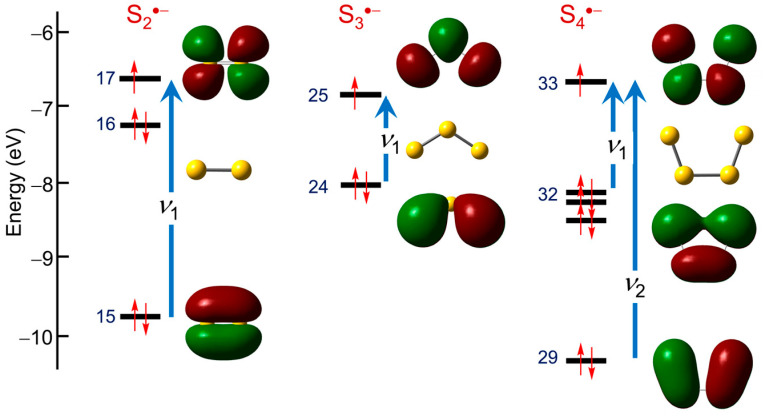
Spin-restricted TD-DFT frontier orbitals and excitations for S_2_^•−^, S_3_^•−^ and cis-S_4_^•−^.

**Figure 8:**
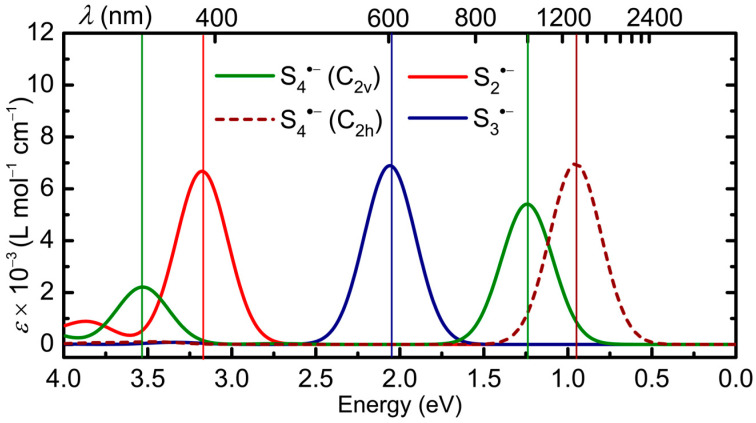
TD-DFT calculated electronic spectra in DMF of S*_n_*^•−^ (*n* = 2, 3, 4), with HWHM = 0.18 eV; band assignments in Table 1.

The TD-DFT calculations refine the qualitative predictions of the HMO model, confirming the nature of the expected transitions (Figure 7) and affording numerical estimates for the *π* → π* excitation energies involved for both the cis (C_2v_) and trans (C_2h_) isomers. Calculated spectra for S*_n_*^•−^ (*n* = 2–4) are shown in Figure 8.

The close correspondence between experimental *λ*_max_ values for *n* = 2 (~400 nm) [18] and *n* = 3 (615–620 nm in DMF or HMPA) [73] and those predicted by TD-DFT (Table 1) provide strong support for the choice of functional, and hence confidence in the calculated values for *n* = 4. The results for *n* = 4 are also in good qualitative agreement with those reported earlier [48,49], and thus help clarify some of the controversies surrounding the spectrophotometric identification of putative S_4_^•−^ species. For both the cis and trans isomers, the first transition (*ν*_1_, 32*β* → 33*β*) lies at or beyond the edge of the visible region (998 nm and 1300 nm, respectively), and for the cis isomer the second (*ν*_2_, 29*β* → 33*β*) is predicted to have *λ*_max_ = 351 nm, placing it relatively close to S_2_^•−^ and also many closed-shell dianionic species, e.g., S_3_^2−^ [42], as well as other radical anions, e.g., S_5_^•−^ (vide infra), from which it would be hard to distinguish. For the trans isomer, the second transition (*ν*_2_, 29*β* → 33*β*) is symmetry-forbidden and has zero oscillator strength (*f* = 0). It will therefore display no signature at all in the visible region, regardless of its concentration in solution. In this light, assertions that S_4_^•−^ has “never been observed” [39,43] by time-resolved spectroelectrochemistry perhaps deserve a second thought; absence of evidence is not evidence of absence.

#### 2.2.2. S_5_^•−^

**Figure 9:**
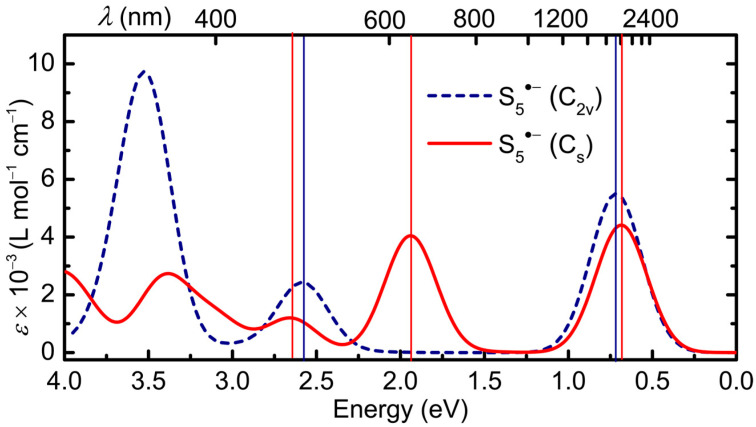
Calculated electronic spectrum of S_5_^•−^ in DMF, in C_2v_ and C_s_ symmetry, with HWHM = 0.18 eV; band assignments in Table 1.

TD-DFT analysis of the optical properties of S_5_^•−^, using the coordinates of the chair-shaped C_s_ structure identified above as the most stable in polar media, affords an electronic spectrum (Figure 9) consisting of a series of bands spread across the entire visible and near-IR regions. However, in contrast to the three short-chain anions already discussed, the chair geometry of S_5_^•−^ is not planar (although the molecule is bisected by a mirror plane), as a result of which rigorous characterization of individual orbitals and excitations between them according to their reflection symmetry in that plane, the classical *σ*/*π* classification, is no longer possible.

**Figure 10:**
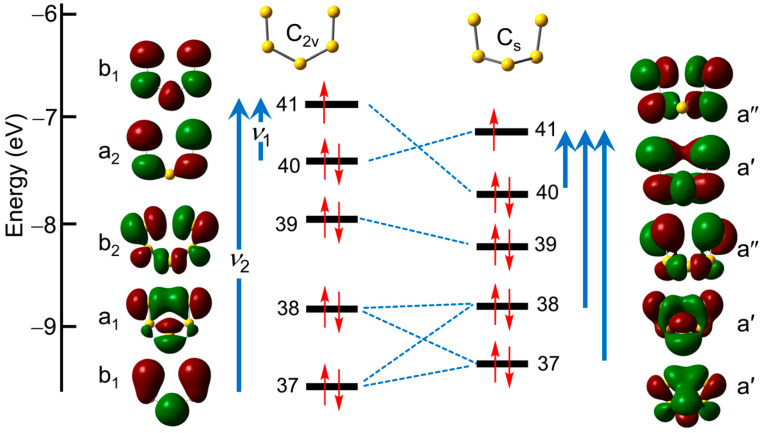
Correlation of spin-restricted TD-DFT frontier orbitals and electronic excitations for S_5_^•−^ in C_2v_ and C_s_ symmetry.

To resolve this difficulty, we examined the orbital make-up and electronic excitations found for the hypothetical planar variation with C_2v_ symmetry. While it is considerably less stable than the C_s_ form, by virtue of increased lone-pair repulsions, its higher symmetry allows for a clearer evaluation of its spectral signature, particularly in relation to the HMO open-chain model developed above. Indeed, it is immediately apparent by inspection of the frontier orbitals illustrated in Figure 10 that excitations 40*β* → 41*β* and 38*β* → 41*β* listed in Table 1 correspond to the two lowest energy *π* → *π* excitations *ν*_1_ and *ν*_2_ predicted by the HMO linear chain model with *N* = 5 (Figure 6). As expected, *ν*_1_ lies deep into the near-IR (*λ*_max_ = 1732 nm), extending the shift to lower energy seen in cis and trans S_4_^•−^ (*λ*_max_ = 998 and 1299 nm, respectively), with *ν*_2_ likewise red-shifted to *λ*_max_ = 478 nm (from 351 nm in cis S_4_^•−^). The third, very intense excitation, from 39*β* → 42*β*, with *λ*_max_ = 352 nm, is not related to the chain model, nor even to a *π* → *π* transition, but is rather a lone-pair *σ* → *σ* process arising from the artificially enforced planarity of the structure.

With this information in hand, the origin of the optical signature of the C_s_ form emerges. The three lowest-lying states can each be described in terms a single dominant excitation from one of the doubly occupied molecular orbitals to the singly occupied molecular orbital (SOMO), that is, the lowest unoccupied molecular orbital (LUMO) for the unrestricted *β*-spins listed in Table 1. Moreover, correlation of the orbitals for the C_2v_ and C_s_ geometries confirms that the HMO chain model still applies, albeit more loosely because of the loss of planarity and consequent *σ*/*π* mixing. Thus, while the ordering of orbitals 40 and 41 is reversed, the first excitation, 40*β* → 41*β* (*λ*_max_ = 1810 nm) can be considered a *quasi- π* → *π* transition related to *ν*_1_ in the HMO model. The next two, 38*β* → 41*β* (*λ*_max_ = 638 nm) and 37*β* → 41*β* (*λ*_max_ = 467 nm), also involve heavily hybridized orbitals, but both are *quasi- π* → *π* processes that can be traced back to *ν*_2_. The higher energy (>3 eV) absorptions comprise a series of less well-defined states arising from multiple excitations (see Table S2).

#### 2.2.3. S_6_^•−^

Addressing the optical properties of the S_6_^•−^ anion presents a quandary. The crystallographic evidence indicates a symmetric chair structure with C_2h_ symmetry, while DFT optimizations point towards a distorted C_2_ version. There are merits to both positions. In solution, and in the absence of environmental constraints, the lower-symmetry C_2_ geometry is probably preferred, but the high space group symmetry of the [Ph_4_P][S_6_] salt appears to hold the chair in the higher-symmetry C_2h_ form. In that light we have performed TD-DFT calculations on both options, using geometries taken from the respective structural optimizations.

**Figure 11:**
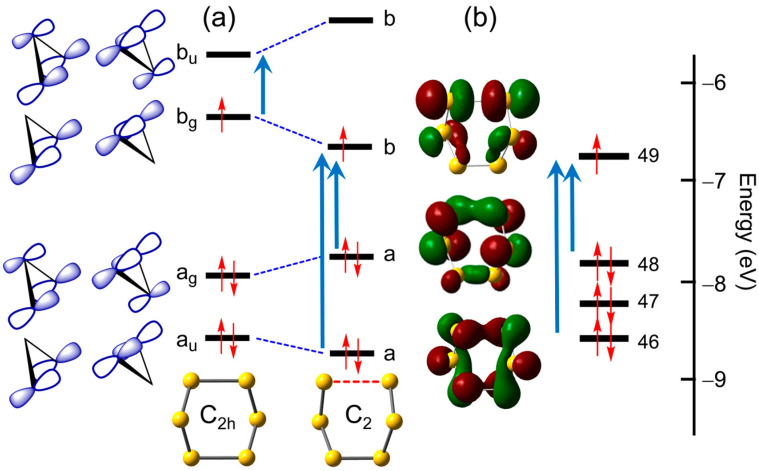
(**a**) Sketch of frontier orbital energies following distortion of S_6_^•−^ from C_2h_ to C_2_ symmetry. (**b**) Spin-restricted TD-DFT frontier orbitals and electronic excitations for S_6_^•−^ in C_2_ symmetry.

As a first step, however, we focus on a qualitative model for describing the two elongated bonds in the symmetric structure. Building on the ideas developed earlier by Dehnicke and coworkers [47], Figure 11a illustrates the two strongly coupled *σ* and σ* orbitals arising from combinations of two S_3_ fragments. A second-order Jahn–Teller distortion from C_2h_ to C_2_ will give rise to mixing of the b_g_ SOMO and b_u_ LUMO, and a widening of the energy gap between them. Figure 11b refines this model, by showing the relevant spin-restricted Kohn–Sham orbitals and eigenvalues for the C_2_ structure, that is, two heavily hybridized, but basically S–S *σ*-bonding, occupied orbitals (46 and 48), and a more localized σ*-orbital (49).

**Figure 12:**
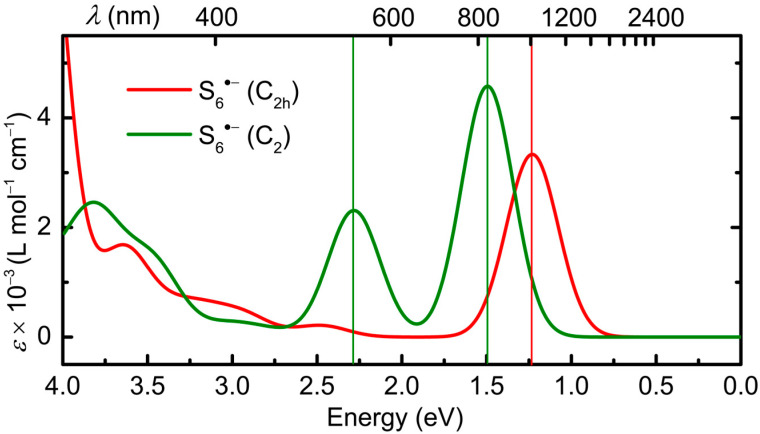
Calculated electronic spectrum of S_6_^•−^ in DMF, in C_2h_ and C_2_ symmetry, with HWHM = 0.18 eV; band assignments in Table 1.

Given this conceptual framework, the optical signatures predicted for the two geometries are readily explained. As shown in Figure 12, the C_2h_ structure displays a single well-resolved band with *λ*_max_ = 1007 nm, which corresponds *not* to electron promotion from the HOMO to the SOMO, which is symmetry-forbidden (*g* → *g*) in C_2h_, but rather to the SOMO-to-LUMO excitation shown in Figure 11a (49*α* → 50*α*, Table 1). In addition, there are a series of less well-defined states that give rise to a broad absorption that extends into the UV region.

For the distorted C_2_ symmetry structure, the excited state manifold is quite different. Two bands are predicted in the visible and near-IR region (*λ*_max_ = 543 nm and 830), which arise primarily from the (now allowed) excitations, 46*β* → 49*β* and 48*β* → 49*β* (Table 1), from occupied orbitals to the SOMO, both of which are essentially *σ* → σ* processes. As in the case of the C_2h_ geometry, there is a broad band extending into the UV region associated with a series of higher energy but less well-defined states.

In summary, the optical properties of both variations of the S_6_^•−^ radical anion are associated with transitions associated with the lengthened S–S *σ*-bonds. In both its excited and ground states, the S_6_^•−^ radical anion behaves like a cyclic molecule.

#### 2.2.4. S_7_^•−^ and S_8_^•−^

The two largest radical anions (*n* = 7 and 8) are the easiest to analyze, as the structural perturbations occasioned by addition of an electron to the parent homocycles are small. Based on the structural parameters provided by the DFT optimizations of the chair and boat conformers of S_7_^•−^, both of Cs symmetry, and of the C_2_ distorted crown geometry of S_8_^•−^, all three rings experience a lengthening of one of the S–S bonds, an effect which can best be described in terms of the formation of a largely localized 2c-3e *σ*-bond.

**Figure 13:**
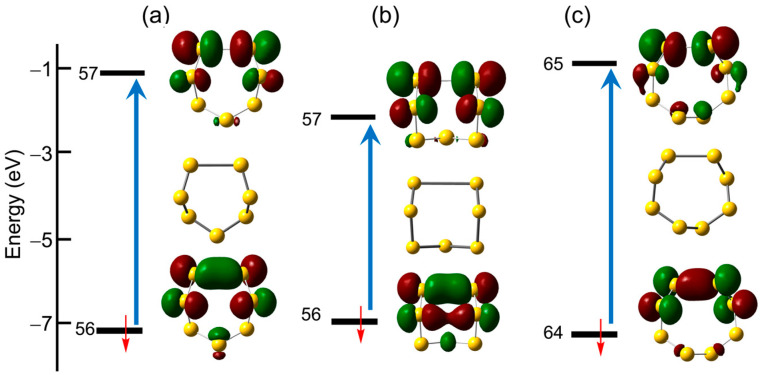
Unrestricted TD-DFT frontier orbitals and calculated *σ* → σ* electronic excitations found for the (**a**) chair and (**b**) boat conformations of S_7_^•−^ and (**c**) the C_2_-distorted crown structure of S_8_^•−^.

**Figure 14:**
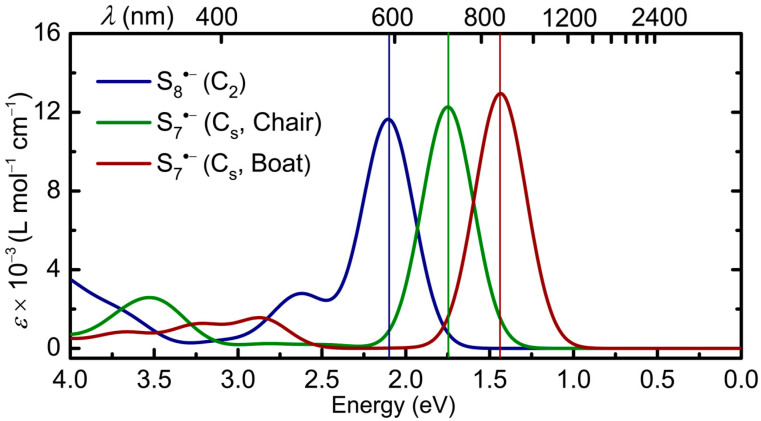
Calculated electronic spectra in DMF of S_7_^•−^ (chair and boat conformations) and S_8_^•−^, with HWHM = 0.18 eV; band assignments in Table 1 and Table S2.

The TD-DFT calculations reinforce this picture, providing a description for the first excited state which involves promotion of an electron between the associated *σ-* and σ*-orbitals of the 2c-3e manifold, that is, the *β*-spin HOMO and LUMO of the two conformers of S_7_^•−^ (56*β* → 57*β*) and those of S_8_^•−^ (64*β* → 65*β*) shown in Figure 13. These transitions give rise to single bands with large oscillator strength in the low-energy visible or near-IR region (Figure 14). As expected, there is a notable difference between the band maxima of the chair (*λ*_max_ = 709 nm) and boat (*λ*_max_ = 864 nm) conformations of S_7_^•−^ which can be traced back to the longer S···S separation found in the latter (Figure 4).

The higher transition energy predicted for S_8_^•−^ (*λ*_max_ = 589 nm) can be attributed to a similar effect, the shorter S···S separation stemming from the mutual staggering of the neighboring bonds and consequent relief from the effects of lone-pair repulsion. Secondary, less intense absorptions, with *λ*_max_ = 474 nm (S_8_^•−^), 358 nm (S_7_^•−^, chair) and 435 nm (S_7_^•−^, boat), are also predicted. These are associated with poorly defined, higher-lying states (Table S2), but their presence may aid in identification. That being said, the strong low energy *σ* → σ* excitation, which dominates the spectrum of all three species, represents the most distinguishing optical feature.

## 3. Discussion

As indicated in the introduction, there are considerable differences of opinion as to when and where the radical anions S*_n_*^•−^ (*n* = 4–8) might be found. While their participation in the multistep redox equilibria associated with the operation of sulfide/polysulfide photoconductor cells, alkali-metal/sulfur batteries, as well as in many biological and organic transformations, is frequently implied, their identification in these complex systems has, not surprisingly, proved elusive. Equally, extensive and detailed spectroscopic and spectroelectrochemical studies, utilizing a wide range of techniques (optical, IR, Raman, EPR spectra), have been unable to provide decisive answers.

The purpose of the present work has been two-fold. Firstly, the most stable structures of the putative radical anions S*_n_*^•−^ (*n* = 4–8) in polar solvents have been identified using high-level DFT methods. Secondly, TD-DFT calculations, performed on the most stable structural candidates, have been used to map out the number, nature and energies of the photochemically accessible excited states for these species. Critical to the validity of this latter step was the ability to assess the numerical reliability of the methods used (choice of LC-functional for TD-DFT work) by comparison of the predicted excitation energies with experimental values for the well-known radical anions S_2_^•−^ and S_3_^•−^. Even so, we neither expect nor claim that the present TD-DFT calculated transition energies will provide a perfect match with experiment for the larger members of the series, especially for the low-energy (near-IR and beyond) excitations. Taken together, however, the results on the entire series of anions S*_n_*^•−^ (*n* = 2–8) provide a frame of reference for distinguishing between different members of the family. Equally important, from an interpretational viewpoint, has been the use of the classical one-electron HMO chain model [74,75] to anticipate both the number and *approximate* energies of *π* → *π* transitions, again using S_2_^•−^ and S_3_^•−^ as reference points. The TD-DFT results suggest that the calculated spectra for S_4_^•−^, and even S_5_^•−^, can be effectively rationalized by this approach. By contrast, the low energy excitations predicted for the essentially cyclic structures of S*_n_*^•−^ (*n* = 6–8) are best described in terms of *σ* → σ* processes within a relatively localized 2c-3e manifold.

**Scheme 2:**
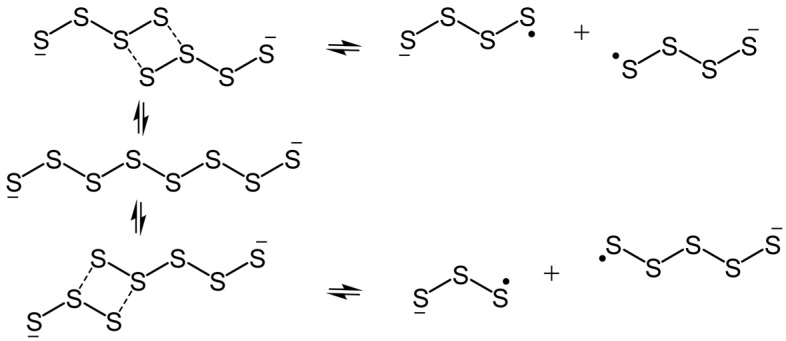
Symmetric and asymmetric dissociation of S_8_^2−^ to the radical anions S*_n_*^•−^ (*n* = 3, 4, 5).

The availability of this information opens the door to the design of experimental strategies for the generation, observation and perhaps even isolation of the radical anions S*_n_*^•−^ (*n* = 4–8). We begin by considering the seminal 1991 report by Rauchfuss and coworkers on the structure and spectroscopic properties of the open-chain octasulfide dianion S_8_^2−^ in the absence of counterion pairing effects [48]. As noted earlier, these authors attributed the strong band with *λ*_max_ = 618 nm that emerged upon dilution of a solution of [Mn(*N*-MeIm)_6_][S_8_] (*N*-MeIm = *N*-methylimidazole) in *N*-MeIm to the presence of the radical anion S_3_^•−^. At the time they rationalized the generation of S_3_^•−^ in terms of a disproportionation of S_8_^2−^ to ¼S_8_ and S_6_^2−^, and subsequent dissociation of the latter, following the conventional interpretation of the electrochemistry community [35,49]. Other radical anions, notably S*_n_*^•−^ (*n* = 4, 5), were not included in the analysis, in part because their optical signatures were unknown. Given the present TD-DFT results, however, the potential involvement of these seemingly missing radical anions can be examined. In particular, we consider the possibility that both might be formed, along with S_3_^•−^, by either symmetric or asymmetric dissociation of the S_8_^2−^ dianion, as indicated in Scheme 1. In addition to the overall thermodynamics of such processes [43], mechanistic considerations may also be important—how easy is it to rupture the distinct S–S bonds along the chain? In response to this question, we suggest that dissociation may proceed via four-center intermediates, as illustrated in Scheme 2. Indeed, in the case of symmetric dissociation, an example of a four-center *π*-dimer has been characterized crystallographically [21].

**Figure 15:**
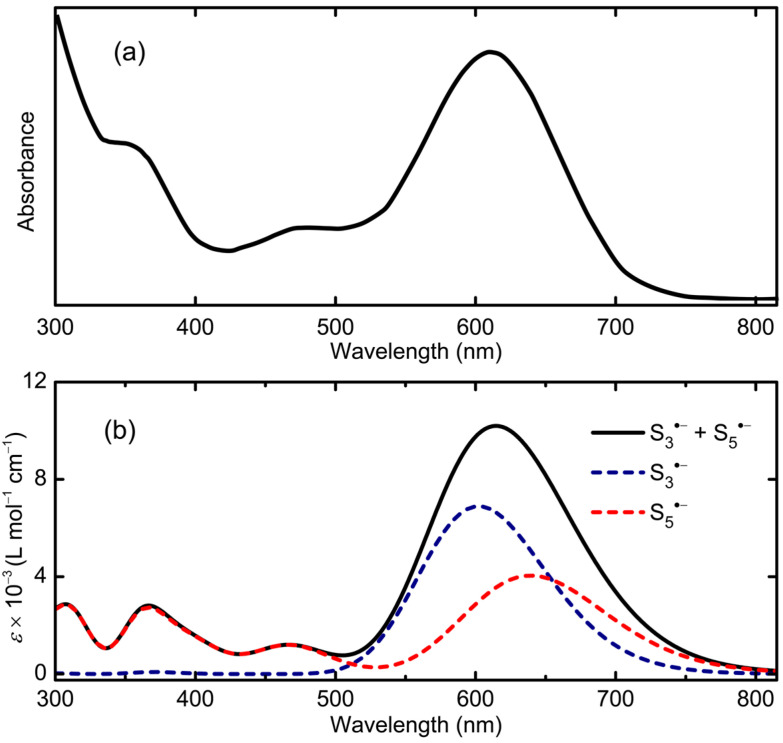
(**a**) Optical spectrum of 0.001 M solution of [Mn(*N*-MeIm)_6_][S_8_] in *N*-MeIm, reproduced from [48]; copyright American Chemical Society. (**b**) Calculated composite spectrum, with HWHM = 0.18 eV, for equally weighted mixture of S_3_^•−^ and S_5_^•−^ in DMF.

As an example of the spectroscopic ramifications of this interpretation, we compare in Figure 15 the experimental spectrum for the highly diluted solution of S_8_^2−^, as reported by Rauchfuss, with an equally weighted composite of the TD-DFT calculated spectra for S_3_^•−^ and S_5_^•−^, the two products of an *asymmetric* dissociation. In the mid-range visible region, the correspondence is remarkable, not only in terms of the overlap and coalescence of the two bands calculated for *n* = 3, 5, but also the presence of the weaker band near 480 nm which, on the present basis, may be assigned to *n* = 5 (calculated *λ*_max_ = 467 nm). Below 400 nm the match is less than ideal, but could be improved by inclusion into the composite of cis S_4_^•−^ (calculated *λ*_max_ = 351 nm), the unique *symmetric* dissociation product. Alternatively, these higher energy absorptions may arise from *un*dissociated S_6_^2−^. That being said, the absence of bands attributable to S_2_^•−^ or S_6_^•−^ suggests dissociation of S_8_^2−^ into these species does not occur to any great extent.

By itself, this single spectral deconvolution exercise does not constitute proof for multiple dissociation pathways, but critical support for the concept could be readily achieved by inspection of the near-IR region of dilute solutions of S_8_^2−^, where one or both of the low-energy (*ν*_1_) bands of S_4_^•−^ and S_5_^•−^ should be present. Moreover, similar spectroscopic analysis at high dilution of solutions of salts of the known hepta- and hexasulfide dianions S_7_^2−^ and S_6_^2−^ should reveal predicable patterns of radical anions, the former affording S_3_^•−^ and S_4_^•−^ (but possibly not S_5_^•−^) and the latter specifically S_3_^•−^; indeed, for S_6_^2−^ this result has already been confirmed [48]. In the same way, observation of the cyclic radical anions S*_n_*^•−^ (*n* = 6–8) may be possible, by examination of highly diluted solutions of long chain dianions such S_10_^2−^ [49] and S_12_^2−^ [50].

In addition to routes to the radical anions which rely solely on dissociative equilibria between radical anions and closed-shell dianions, direct chemical synthesis may be possible, as in the case of the radical anion salt [Ph_4_P][S_6_], which was prepared by the rather unusual reaction of H_2_S and Me_3_SiN_3_ in the presence of [Ph_4_P][N_3_] [52]. If this procedure could be adapted to incorporate the use of other bulky cations, e.g., PPN^+^, different crystal morphologies might be generated. That being the case, would the structure of the resulting anion be constrained to C_2h_ symmetry, as in [Ph_4_P][S_6_], or display the more stable distorted C_2_ shape? Even in the solid state, the expected optical signatures (Figure 12) are predicted to be quite different. Alternatively, not only S_6_^•−^ but also S_7_^•−^ and S_8_^•−^ could be accessible by electrochemical reduction of the appropriate neutral allotrope [76,77,78]. The latter two anions have very distinct optical profiles, although we add the caveat that the footprint of S_8_^•−^ may easily be confused with that of S_3_^•−^.

Chemical reduction methods, for example using organometallic reducing agents such as cobaltocene, which is known to afford salts of closed [79] and open shell anions [80] with sulfur-based electron acceptors, may also provide access to salts of S_8_^•−^. In this connection Woollins et al. obtained the salt [Cp_2_Co][S_3_N_3_] from the reaction of S_4_N_4_ with cobaltocene in THF [81]. This transformation probably involves the initial generation of the radical anion S_4_N_4_^•−^, known from electrochemical studies to be formed by one-electron reduction of S_4_N_4_, which then undergoes ring contraction to produce S_3_N_3_^−^ [77].

Just as chemical oxidation of cyclo-S_8_ has afforded the radical cation S_8_^•+^ [78], treatment of salts of the dianions S_7_^2−^ [82] and S_8_^2−^ [47,48] with mild oxidants, e.g., iodine or *N*-bromosuccinimide, might well yield the corresponding radical anions S_7_^•−^ and S_8_^•−^. An alternative to chemical oxidation is the use of photolysis to generate polysulfide radical anions from the corresponding dianions. This approach is based on the recent work of Chiba and co-workers on the production of polysulfide radical anions S*_n_*^•−^ (*n* = 3, 4) [25,26,27] via photolysis of the polysulfide dianion S_4_^2−^, as well as on insights provided by investigations into the photoelectrochemical oxidation of S^2−^ by metal-sulfide quantum dots [14,15].

Last, but not least, we acknowledge the role that serendipity has played in the advancement of the chemistry of polysulfide radical anions. For example, the procedures used to achieve the isolation and characterization of S_6_^•−^ [52] and of the *π*-dimer of S_4_^•−^ [21] would have been difficult to predict a priori, but their somewhat fortuitous discovery strengthens the conviction that continued exploration will yield new insights. Towards that end the present results may prove useful.

## 4. Computational Methods

Unrestricted density functional theory (DFT) calculations were performed with the Gaussian 16 suite of programs [74], using the default ultrafine integration grids. Geometry optimizations employed the hybrid-adapted Perdew–Burke–Ernzerhof functional (PBE0) [75,83] and Ahlrichs’ quadruple-*ξ* valence def2-QZVP basis set [84], without additional diffuse functions [85] but with Grimme’s empirical correction (D3) [81,86] included to account for possible dispersion effects [87]. For most anions several geometries, both cyclic and acyclic, were considered, and wherever a stationary point was located a full vibrational analysis was performed to determine whether or not it corresponded to a true energy minimum. Preferred geometries were further optimized with the inclusion of the polarized continuum model (PCM) [88] to account for the effects of solvation, dimethylformamide (DMF) being set as a representative polar solvent. Listings of total energies, vibrational frequencies and cartesian coordinates, with and without PCM, are provided in the SI.

The optical properties of the polysulfide radical anions were explored using single point unrestricted time-dependent (TD) DFT calculations, with the same def2-QZVP basis set and PCM included. The use of several long range corrected (LC) functionals, which are known to provide reasonable estimates of low energy (charge transfer, Rydberg-like) excitations in molecular species [89,90], including radicals [91] and sulfur-containing radical anions [92], was explored. The best results, reported here, employed the empirical dispersion-corrected density functional *ω*B97XD [93], which is well recognized for its overall performance [94]. All tabulated excitation energies refer to spin-unrestricted calculations, but for ease of visualization some of the orbital energy diagrams are based on spin-restricted wavefunctions. All spectral plots, prepared using Gaussview 6 [95], employed Gaussian band shapes with the half-width at half maximum (HWHM) value set at 0.18 eV. The associated extinction coefficients were derived using routines available within Gaussview. Kohn–Sham wavefunctions were also plotted using Gaussview.

## 5. Conclusions

The DFT and TD-DFT calculations reported here represent the first comprehensive attempt both to predict and to rationalize the optical properties of the entire family of polysulfide radical anions S*_n_*^•−^ (*n* = 2–8). Our results confirm earlier predictions [52,53] that the first *π* → *π* transition for both the cis (C_2v_) and slightly less stable trans (C_2h_) isomers of S_4_^•−^ should occur in the near-IR region. However, a second *π* → *π* transition at around 350 nm is expected for the cis isomer of S_4_^•−^. Based on Seel’s early results [19,20], a band near 490 nm has often been attributed to this species, but these conclusions are questionable [3]. At least in dilute solution this band may originate from S_5_^•−^, the most stable form of which possesses an acyclic structure with C_s_ symmetry, and is predicted to display three optical absorption bands, two in the visible and one in the near-IR region.

The S_6_^•−^ radical anion is an interesting and unique example of a polysulfide radical anion that has been structurally characterized in the solid state. In the ion-separated salt [Ph_4_P][S_6_] the anion displays a cyclic structure (C_2h_ symmetry) with two long S–S bonds [51], while DFT geometry optimization points to a distorted cyclic structure (C_2_ symmetry) with one long S–S bond as a more stable arrangement. The predicted electronic spectra for these two forms are very different, with *λ*_max_ = 1007 nm vs. 830 and 543 nm, respectively.

To date the heptasulfide radical anion S_7_^•−^ has received scant attention, but the present DFT results point to a cyclic structure with two energetically similar conformers, chair and boat (Cs symmetry), both displaying one long S−S bond best described in terms of a localized 2c-3e *σ*-interaction. Electronic excitation within this manifold gives rise to a strong visible/near-IR absorption with calculated values of *λ*_max_ = 709 nm and 864 nm for chair and boat, respectively. The octasulfide radical anion S_8_^•−^, which carries particular significance as the initial product of the electrochemical reduction of cyclo-S_8_ [3], is also predicted to possess a distorted cyclic structure (C_2_ symmetry) exhibiting, like S_7_^•−^, a single elongated 2c-3e S−S bond. The associated *σ* → σ* excitation generates a strong visible absorption band with a calculated *λ*_max_ = 589 nm.

## Figures and Tables

**Table 1:** TD-DFT electronic excitations for S*_n_*^•−^ (*n* = 2–8).

*n* in S*_n_*^•−^	State	Excitation *^a^*	State	*ν,* eV	*λ*, nm	*f*
2 (D_∞h_)	^2^Σ_g_	15*β* → 17*β*	^2^Σ_u_	3.177	390.3	0.0891
3 (C_2v_)	^2^B_1_	24*β* → 25*β*	^2^B_2_	2.059	602.1	0.0920
4 (C_2v_)	^2^A_2_	32*β* → 33*β*	^2^B_2_	1.242	998.3	0.0722
	^2^A_2_	29*β* → 33*β*	^2^A_1_	3.533	351.0	0.0286
4 (C_2h_)	^2^B_g_	32*β* → 33*β*	^2^B_u_	0.954	1299.5	0.0930
	^2^B_g_	29*β* → 33*β*	^2^A_g_	3.578	346.6	0.0000 *^b^*
5 (C_2v_)	^2^B_1_	40*β* → 41*β*	^2^B_2_	0.716	1732.3	0.0734
	^2^B_1_	38*β* → 41*β*	^2^A_1_	2.583	479.9	0.0332
	^2^B_1_	39*β* → 42*β*	^2^A_1_	3.519	352.4	0.1258
5 (C_s_)	^2^A″	40*β* → 41*β*	^2^A″	0.685	1809.6	0.0589
	^2^A″	38*β* → 41*β*	^2^A″	1.940	638.2	0.0540
	^2^A″	37*β* → 41*β*	^2^A″	2.653	467.4	0.0158
6 (C_2h_)	B_g_	49*α* → 50*α*	A_u_	1.231	1007.3	0.0445
	B_g_	48*β* → 49*β*	B_g_	1.978	627.5	0.0000 *^b^*
6 (C_2_)	^2^B	48*β* → 49*β*	^2^B	1.495	829.6	0.0611
	^2^B	46*β* → 49*β*	^2^B	2.283	543.1	0.0308
7 (C_s_ chair)	^2^A″	56*β* → 57*β*	^2^A″	1.749	708.7	0.1637
7 (C_s_ boat)	^2^A″	56*β* → 57*β*	^2^A″	1.435	863.9	0.1729
8 (C_2_ crown)	^2^B	64*β* → 65*β*	^2^B	2.104	589.3	0.1551

*^a^* Dominant spin-orbital transitions from unrestricted TD-U*ω*B97X-D/PCM/def2-QZVP calculations, with PCM = DMF. *^b^* Electric dipole forbidden.

## Data Availability

Details of the computational data are in the Supplementary Materials.

## Supplementary Materials

The following supporting information (21 pages total) can be downloaded at: https://www.mdpi.com/article/10.3390/molecules28155654/s1, Figure S1: Optimized geometrical parameters; Table S1: Total electronic energies; Table S2: Excitation energies, oscillator strengths and orbital contributions; Tables S3–S11: Gaussian archive entries; Tables S12–S17: Frequency calculations.
