# Supplementary material for: Structures and Spectroscopic Properties of Polysulfide Radical Anions: A Theoretical Perspective
Source: Molecules. 2023 Jul 26;28(15):5654. doi: 10.3390/molecules28155654 (PMC10419630; doi:10.3390/molecules28155654)
Supplement: Supplementary file 1 [file molecules-28-05654-s001.zip › molecules-2487437-supplementary.pdf]

Supporting Information

Structures and Spectroscopic Properties of Polysulfide Radical Anions: A Theoretical Perspective

Tristram Chivers[1]* and Richard T. Oakley[2]*

[1]*Department of Chemistry, University of Calgary, Calgary, Alberta T2N 1N4, Canada*
[2]*Department of Chemistry, University of Waterloo, Waterloo, Ontario N2L 3G1, Canada*

*E-mail: chivers@ucalgary.ca (TC) and oakley@uwaterloo.ca (RTO)

Contents (21 pages total)

**Figure S1** Optimized geometrical parameters in gas phase and with PCM	Page S2
**Table S1** Total electronic energies for $S_n^{\bullet-}$ (n = 2 - 8)	Page S3
**Table S2** Excitation energies, oscillator strengths and orbital contributions	Pages S4-S6
**Table S3** Gaussian archive entries for $S_2^{\bullet-}$ and $S_3^{\bullet-}$	Page S7
**Table S4** Gaussian archive entries for $S_4^{\bullet-}$	Page S8
**Table S5** Gaussian archive entries for $S_4^{\bullet-}$ and $S_5^{\bullet-}$	Page S9
**Table S6** Gaussian archive entries for $S_5^{\bullet-}$	Page S10
**Table S7** Gaussian archive entries for $S_6^{\bullet-}$	Page S11
**Table S8** Gaussian archive entries for $S_6^{\bullet-}$ and $S_7^{\bullet-}$	Page S12
**Table S9** Gaussian archive entries for $S_7^{\bullet-}$	Page S13
**Table S10** Gaussian archive entries for $S_8^{\bullet-}$	Page S14
**Table S11** Gaussian archive entries for $S_8^{\bullet-}$	Page S15
**Table S12** Frequency calculations on $S_4^{\bullet-}$ optimizations	Page S16
**Table S13** Frequency calculations on $S_5^{\bullet-}$ optimizations	Page S17
**Table S14** Frequency calculations on $S_6^{\bullet-}$ optimizations	Page S18
**Table S15** Frequency calculations on $S_7^{\bullet-}$ optimizations	Page S19
**Table S16** Frequency calculations on $S_7^{\bullet-}$ and $S_8^{\bullet-}$ optimizations	Page S20
**Table S17** Frequency calculations on $S_8^{\bullet-}$ optimizations	Page S21

Figure S1 Optimized geometrical parameters in gas phase and with PCM

Numbers in regular text (black) are from UPBE0/D3/def2-QZVP gas phase optimizations. Numbers in parenthesis (red) refer to UPBE0/D3/def2-QZVP optimizations with (PCM=DMF) runs.

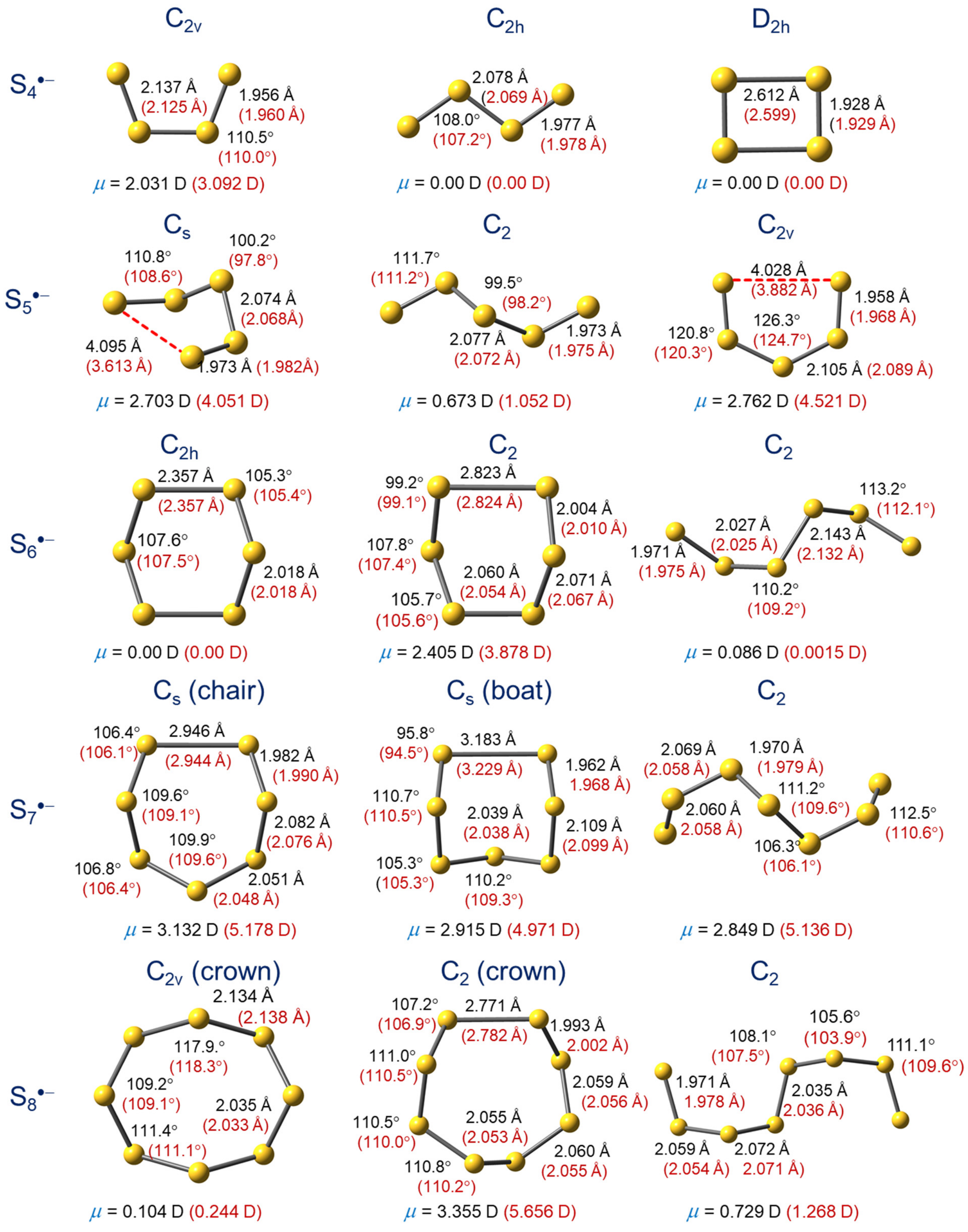

Table S1 Total electronic energies for $S_n^{\bullet-}$ (n = 2 - 8)

Method 1: (U)PBE0/D3/Def2-QZVP optimization
Method 2: (U)PBE0/D3/PCM/Def2-QZVP optimization with PCM = DMF

**Anion**	**Geometry**	**Method 1**		**Method 2**	
		E_{tot} **(H)**	E_{rel} **(kJ mol^{-1})**	E_{tot} **(H)**	E_{rel} **(kJ mol^{-1})**
$S_2^{\bullet-}$	$D_{\infty h}$	-796.19689196		-796.28981245	
$S_3^{\bullet-}$	C_{2v}	-1194.31425530		-1194.39732054	
$S_4^{\bullet-}$	C_{2v}	-1592.40394686	0.00	-1592.48316274	0.00
$S_4^{\bullet-}$	C_{2h}	-1592.40443155	-1.27	-1592.47941766	9.83
$S_4^{\bullet-}$	D_{2h}	-1592.39459386	24.56	-1592.47161007	30.33
$S_5^{\bullet-}$	C_s	-1990.49240983	0.00	-1990.56727523	0.00
$S_5^{\bullet-}$	C_2	-1990.49239966	0.03	-1990.56242578	12.73
$S_5^{\bullet-}$	C_{2v}	-1990.46601665	69.30	-1990.54011035	71.32
$S_6^{\bullet-}$	C_{2h}	-2388.57866510	22.51	-2388.64842243	27.11
$S_6^{\bullet-}$	C_2 chair	-2388.58724020	0.00	-2388.65874675	0.00
$S_6^{\bullet-}$	C_2 chain	-2388.58760293	-0.95	-2388.65349016	13.80
$S_7^{\bullet-}$	Cs chair	-2786.68613154	0.00	-2786.75475259	0.00
$S_7^{\bullet-}$	C_2 boat	-2786.68401462	5.56	-2786.75341901	3.50
$S_7^{\bullet-}$	C_2 chain	-2786.67605005	26.47	-2786.74027237	38.02
$S_8^{\bullet-}$	C_{2v} crown	-3184.77471500	20.60	-3184.83755865	28.62
$S_8^{\bullet-}$	C_2 crown	-3184.78255934	0.00	-3184.84845949	0.00
$S_8^{\bullet-}$	C_2 chain	-3184.77026941	32.27	-3184.83195815	43.32

Table S2 Excitation energies, oscillator strengths and orbital contributions.

From single point TD-UωB97XD/def2-QZVP (PCM=DMF) runs, with geometries taken from UPBE0/D3/def2-QZVP (PCM=DMF) optimizations.

$S_2^{\bullet-}$ radical anion in $D_{\infty h}$ symmetry

```
Excited State   2:  1.996-SGU    3.1769 eV  390.27 nm  f=0.0891  <S**2>=0.746
     15B -> 17B         0.98226
```

$S_3^{\bullet-}$ radical anion in C_{2v} symmetry

```
Excited State   3:  2.004-B2     2.0591 eV  602.14 nm  f=0.0920  <S**2>=0.754
     24B -> 25B         0.98594
```

$S_4^{\bullet-}$ radical anion in C_{2v} symmetry

```
Excited State   1:  2.002-B2     1.2420 eV  998.30 nm  f=0.0722  <S**2>=0.752
     31A -> 34A         0.14900
     31B -> 34B         0.11138
     32B -> 33B         0.99023
     32B <- 33B         0.10921
Excited State   8:  2.031-A1     3.5327 eV  350.96 nm  f=0.0286  <S**2>=0.782
     30A -> 34A         0.23376
     29B -> 33B         0.96065
```

$S_4^{\bullet-}$ radical anion in C_{2h} symmetry

```
Excited State   1:  2.001-BU     0.9541 eV 1299.48 nm  f=0.0930  <S**2>=0.751
     31A -> 34A         0.12643
     32B -> 33B         0.99839
     32B <- 33B         0.14441
Excited State   8:  2.016-AG     3.5775 eV  346.57 nm  f=0.0000  <S**2>=0.766
     30A -> 34A         0.13054
     29B -> 33B         0.97995
```

$S_5^{\bullet-}$ radical anion in C_{2v} symmetry

```
Excited State   1:  2.001-B2     0.7157 eV 1732.25 nm  f=0.0734  <S**2>=0.751
     39A -> 42A         0.11472
     40B -> 41B         1.00686
     40B <- 41B         0.19077
Excited State   8:  2.054-A1     2.5833 eV  479.94 nm  f=0.0322  <S**2>=0.805
     38A -> 42A        -0.18566
     39A -> 43A         0.22566
     38B -> 41B         0.94657
Excited State  13:  2.009-B2     3.5186 eV  352.37 nm  f=0.1258  <S**2>=0.759
     39A -> 42A         0.67401
     39B -> 42B         0.71565
     40B -> 41B        -0.12458
```

Table S2 (cont'd). Excitation energies, oscillator strengths and orbital contributions.

From single point TD-UωB97XD/D3/def2-QZVP (PCM=DMF) runs, with geometries taken from UPBE0/D3/def2-QZVP (PCM=DMF) optimizations.

$S_5^{\bullet-}$ radical anion in C_s symmetry

```
Excited State   1:  1.992-A"     0.6851 eV 1809.61 nm  f=0.0589  <S**2>=0.742
     38B -> 41B         0.15204
     40B -> 41B         0.98847
     40B <- 41B         0.13772
Excited State   3:  2.016-A"     1.9397 eV  639.21 nm  f=0.0540  <S**2>=0.766
     38B -> 41B         0.97837
     40B -> 41B        -0.14627
Excited State   4:  2.018-A"     2.6529 eV  467.36 nm  f=0.0158  <S**2>=0.768
     37B -> 41B         0.99220
Excited State   6:  3.137-A"     3.0996 eV  400.00 nm  f=0.0155  <S**2>=2.210
     39A -> 42A         0.11176
     40A -> 43A        -0.39324
     41A -> 42A        -0.43053
     39B -> 43B        -0.11814
     40B -> 42B         0.75729
Excited State   8:  2.077-A'     3.3776 eV  367.08 nm  f=0.0173  <S**2>=0.828
     40A -> 42A        -0.16491
     40A -> 44A        -0.10643
     41A -> 43A        -0.62120
     36B -> 41B         0.64592
     40B -> 43B        -0.33696
Excited State   9:  2.070-A'    3.4362 eV  360.82 nm  f=0.0173  <S**2>=0.822
     40A -> 42A         0.20033
     41A -> 43A         0.48403
     36B -> 41B         0.73208
     40B -> 43B         0.36905
```

$S_6^{\bullet-}$ radical anion in C_{2h} symmetry

```
Excited State   1:  1.991-AU     1.2308 eV 1007.33 nm  f=0.0445  <S**2>=0.741
     49A -> 50A         0.99789
     49A <- 50A         0.12508
Excited State  11:  2.072-BU     3.3234 eV  373.06 nm  f=0.0057  <S**2>=0.824
     46A -> 50A        -0.14764
     49A -> 53A         0.95944
     49A -> 57A        -0.10894
     47B -> 49B        -0.12823
Excited State  13:  2.810-BU     3.6411 eV  340.51 nm  f=0.0212  <S**2>=1.724
     46A -> 50A         0.81317
     48A -> 50A        -0.23972
     49A -> 53A         0.11641
     43B -> 49B         0.16183
     45B -> 50B        -0.15458
     48B -> 50B        -0.45490
```

Table S2 (cont'd). Excitation energies, oscillator strengths and orbital contributions.

From single point TD-UωB97XD/D3/def2-QZVP (PCM=DMF) runs, with geometries taken from UPBE0/D3/def2-QZVP (PCM=DMF) optimizations.

$S_6^{\bullet-}$ radical anion in C_2 symmetry

```
Excited State   1:  2.009-B      1.4946 eV  829.57 nm  f=0.0611  <S**2>=0.759
     45B -> 49B        0.11135
     46B -> 49B        0.15768
     48B -> 49B        0.96994
Excited State   3:  2.014-B      2.2827 eV  543.14 nm  f=0.0308  <S**2>=0.764
     45B -> 49B        0.31320
     46B -> 49B        0.91568
     48B -> 49B       -0.17920
```

$S_7^{\bullet-}$ radical anion in C_2 (chair) symmetry

```
Excited State   1:  2.001-A"     1.7494 eV  708.74 nm  f=0.1637  <S**2>=0.751
     56B -> 57B        0.97968
Excited State   7:  2.809-A"     3.4656 eV  357.75 nm  f=0.0225  <S**2>=1.723
     57A -> 58A        0.26737
     57A -> 62A       -0.23110
     55B -> 60B       -0.13143
     56B -> 59B        0.88297
```

$S_7^{\bullet-}$ radical anion in C_2 (boat) symmetry

```
Excited State   1:  1.996-A"     1.4352 eV  863.85 nm  f=0.1729  <S**2>=0.746
     56B -> 57B        0.98414
Excited State   5:  2.022-A'     2.8513 eV  434.83 nm  f=0.0194  <S**2>=0.772
     50B -> 57B       -0.11398
     53B -> 57B        0.97068
```

$S_8^{\bullet-}$ radical anion in C_2 (crown) symmetry

```
Excited State   1:  2.008-B      2.1041 eV  589.26 nm  f=0.1551  <S**2>=0.758
     65A -> 67A       -0.15189
     62B -> 65B        0.13497
     64B -> 65B        0.95105
     64B -> 66B       -0.11627
Excited State   4:  2.215-B      2.6136 eV  474.38 nm  f=0.0299  <S**2>=0.976
     64A -> 66A       -0.11251
     64A -> 68A       -0.22211
     65A -> 67A        0.80388
     65A -> 70A       -0.10951
     65A -> 71A       -0.17883
     62B -> 65B       -0.38543
     64B -> 65B        0.19873
```

Table S3 Gaussian archive entries for $S_2^{\bullet-}$ and $S_3^{\bullet-}$

UPBE0/D3/Def2-QZVP geometry optimization for $S_2^{\bullet-}$ in $D_{\infty h}$.

```
1|1|UNPC-DESKTOP-3H3V0JT|FOpt|UPBE1PBE|def2QZVP|S2(1-,2)|HOME|24-Apr-2
 023|0||# upbe1pbe/def2qzvp geom=connectivity opt empiricaldispersion=g
 d3||upbe1pbe/D3/def2qzvp S2- radical anion full optimization with GD3|
 |-1,2|S,0.,0.,0.9981817674|S,0.,0.,-0.9981817674||Version=EM64W-G16Rev
 B.01|HF=-796.196892|S2=0.756628|S2-1=0.|S2A=0.750029|RMSD=4.293e-009|R
 MSF=8.026e-007|Dipole=0.,0.,0.|Quadrupole=2.1285549,-0.0342475,-2.0943
 074,0.,0.,0.|PG=D*H [C*(S1.S1)]||@
```

UPBE0/D3/Def2-QZVP geometry optimization for $S_2^{\bullet-}$ in $D_{\infty h}$ with PCM=DMF.

```
1|1|UNPC-DESKTOP-3H3V0JT|FOpt|UPBE1PBE|def2QZVP|S2(1-,2)|HOME|24-Apr-2
 023|0||# opt upbe1pbe/def2qzvp scrf=(solvent=n,n-dimethylformamide,pcm
 ) geom=connectivity empiricaldispersion=gd3||upbe1pbe/D3/def2qzvp S2-
 radical anion full optimization in DMF||-1,2|S,0.,0.,0.99720439|S,0.,0
 .,-0.99720439||Version=EM64W-G16RevB.01|HF=-796.2898124|S2=0.756667|S2
 -1=0.|S2A=0.75003|RMSD=3.095e-009|RMSF=2.241e-005|Dipole=0.,0.,0.|Quad
 rupole=1.986247,-0.26987,-1.7163769,0.,0.,0.|PG=D*H [C*(S1.S1)]||@
```

UPBE0/D3/Def2-QZVP geometry optimization for $S_3^{\bullet-}$ in C_{2v}.

```
1|1|UNPC-DESKTOP-3H3V0JT|FOpt|UPBE1PBE|def2QZVP|S3(1-,2)|HOME|24-Apr-2
 023|0||# opt geom=connectivity def2qzvp upbe1pbe empiricaldispersion=g
 d3||upbe1pbe/D3/def2qzvp S3- radical anion C2v full optimization||-1,2
 |S,0.,-1.6790965754,-0.3520910246|S,0.,1.6790965754,-0.3520910246|S,0.
 ,0.,0.7041663992||Version=EM64W-G16RevB.01|State=2-B1|HF=-1194.3142553
 |S2=0.756108|S2-1=0.|S2A=0.750031|RMSD=9.448e-009|RMSF=4.959e-005|Dipo
 le=0.,0.,0.4003634|Quadrupole=3.6484154,-6.2676459,2.6192305,0.,0.,0.|
 PG=C02V [C2(S1),SGV(S2)]||@
```

UPBE0/D3/Def2-QZVP geometry optimization for $S_3^{\bullet-}$in C_{2v} with PCM=DMF.

```
1|1|UNPC-DESKTOP-3H3V0JT|FOpt|UPBE1PBE|def2QZVP|S3(1-,2)|HOME|24-Apr-2
 023|0||# opt geom=connectivity def2qzvp upbe1pbe empiricaldispersion=g
 d3 scrf=(solvent=n,n-dimethylformamide,pcm)||upbe1pbe/D3/def2qzvp S3-
 radical anion C2v full optimization with PCM (DMF)||-1,2|S,0.,-1.66697
 13629,-0.3577928024|S,0.,1.6669713629,-0.3577928024|S,0.,0.,0.71556995
 48||Version=EM64W-G16RevB.01|State=2-B1|HF=-1194.3973205|S2=0.7561|S2-
 1=0.|S2A=0.750031|RMSD=9.868e-009|RMSF=1.838e-005|Dipole=0.,0.,0.53882
 15|Quadrupole=3.3374583,-5.8190681,2.4816098,0.,0.,0.|PG=C02V [C2(S1),
 SGV(S2)]||@
```

Table S4 Gaussian archive entries for $S_4^{\bullet-}$

UPBE0/D3/Def2-QZVP geometry optimization for $S_4^{\bullet-}$in C_{2v}.

```
1|1|UNPC-DESKTOP-3H3V0JT|FOpt|UPBE1PBE|def2QZVP|S4(1-,2)|HOME|24-Apr-2
 023|0||# opt geom=connectivity def2qzvp upbe1pbe empiricaldispersion=g
 d3||upbe1pbe/D3/def2qzvp S4- radical anion with C2v optimization and D
 3||-1,2|S,0.,1.752493898,-0.9159957794|S,0.,1.0684059939,0.9159957794|
 S,0.,-1.0684059939,0.9159957794|S,0.,-1.752493898,-0.9159957794||Versi
 on=EM64W-G16RevB.01|State=2-A2|HF=-1592.4039469|S2=0.755478|S2-1=0.|S2
 A=0.750027|RMSD=6.257e-009|RMSF=7.018e-005|Dipole=0.,0.,0.7988865|Quad
 rupole=3.9904833,-6.6228027,2.6323194,0.,0.,0.|PG=C02V [SGV(S4)]||@
```

UPBE0/D3/Def2-QZVP geometry optimization for $S_4^{\bullet-}$in C_{2v} with PCM=DMF.

```
1|1|UNPC-DESKTOP-3H3V0JT|FOpt|UPBE1PBE|def2QZVP|S4(1-,2)|HOME|24-Apr-2
 023|0||# opt geom=connectivity def2qzvp empiricaldispersion=gd3 upbe1p
 be scrf=(solvent=n,n-dimethylformamide,pcm)||upbe1pbe/D3/def2qzvp S4-
 radical anion with C2v optimization and PCM (DMF)||-1,2|S,0.,1.7321884
 376,0.9209258568|S,0.,1.0623444266,-0.9209258568|S,0.,-1.0623444266,-0
 .9209258568|S,0.,-1.7321884376,0.9209258568||Version=EM64W-G16RevB.01|
 State=2-A2|HF=-1592.4831627|S2=0.755454|S2-1=0.|S2A=0.750027|RMSD=9.21
 0e-009|RMSF=9.375e-005|Dipole=0.,0.,-1.2163231|Quadrupole=3.5086595,-6
 .3168033,2.8081439,0.,0.,0.|PG=C02V [SGV(S4)]||@
```

UPBE0/D3/Def2-QZVP geometry optimization for $S_4^{\bullet-}$in C_{2h}.

```
1|1|UNPC-DESKTOP-3H3V0JT|FOpt|UPBE1PBE|def2QZVP|S4(1-,2)|HOME|24-Apr-2
 023|0||# opt geom=connectivity def2qzvp upbe1pbe empiricaldispersion=g
 d3||upbe1pbe/D3/def2qzvp S4- radical anion C2h optimization||-1,2|S,2.
 4923813246,-0.2159949453,0.|S,0.7505302839,0.718752841,0.|S,-0.7505302
 839,-0.718752841,0.|S,-2.4923813246,0.2159949453,0.||Version=EM64W-G16
 RevB.01|State=2-BG|HF=-1592.4044316|S2=0.75517|S2-1=0.|S2A=0.750024|RM
 SD=9.916e-009|RMSF=1.451e-004|Dipole=0.,0.,0.|Quadrupole=-12.8086829,6
 .4421333,6.3665496,0.7816953,0.,0.|PG=C02H [SGH(S4)]||@
```

UPBE0/D3/Def2-QZVP geometry optimization for $S_4^{\bullet-}$ in C_{2h} with PCM=DMF.

```
1|1|UNPC-DESKTOP-3H3V0JT|FOpt|UPBE1PBE|def2QZVP|S4(1-,2)|HOME|24-Apr-2
 023|0||# opt geom=connectivity def2qzvp empiricaldispersion=gd3 upbe1p
 be scrf=(solvent=n,n-dimethylformamide,pcm)||upbe1pbe/D3/def2qzvp S4-
 radical anion C2h optimization with PCM (DMF)||-1,2|S,-0.2196403823,2.
 4789785868,0.|S,0.7228921786,0.7400302184,0.|S,-0.7228921786,-0.740030
 2184,0.|S,0.2196403823,-2.4789785868,0.||Version=EM64W-G16RevB.01|Stat
 e=2-BG|HF=-1592.4794177|S2=0.755196|S2-1=0.|S2A=0.750024|RMSD=5.832e-0
 09|RMSF=1.871e-005|Dipole=0.,0.,0.|Quadrupole=6.4813058,-12.5616565,6.
 0803506,0.9820079,0.,0.|PG=C02H [SGH(S4)]||@
```

Table S5 Gaussian archive entries for $S_4^{\bullet-}$ and $S_5^{\bullet-}$

UPBE0/D3/Def2-QZVP geometry optimization for $S_4^{\bullet-}$ in D_{2h}.

1|1|UNPC-DESKTOP-3H3V0JT|FOpt|UPBE1PBE|def2QZVP|S4(1-,2)|HOME|10-May-2
023|0||# opt geom=connectivity def2qzvp empiricaldispersion=gd3 upbe1p
be||upbe1pbe/D3/def2qzvp rectangular S4- radical anion with D2h full o
ptimization||-1,2|S,0.,0.96424717,1.30579883|S,0.,-0.96424717,1.305798
83|S,0.,-0.96424717,-1.30579883|S,0.,0.96424717,-1.30579883||Version=E
M64W-G16RevB.01|State=2-AU|HF=-1592.3945939|S2=0.755016|S2-1=0.|S2A=0.
750023|RMSD=2.158e-009|RMSF=1.608e-004|Dipole=0.,0.,0.|Quadrupole=2.93
27461,2.1681393,-5.1008854,0.,0.,0.|PG=D02H [SG(S4)]||@

UPBE0/D3/Def2-QZVP geometry optimization for $S_4^{\bullet-}$ in D_{2h} with PCM=DMF.

1|1|UNPC-DESKTOP-3H3V0JT|FOpt|UPBE1PBE|def2QZVP|S4(1-,2)|HOME|24-Apr-2
023|0||# opt geom=connectivity def2qzvp upbe1pbe scrf=(solvent=n,n-dim
ethylformamide,pcm)||upbe1pbe/D3/def2qzvp rectangular S4- radical anio
n with D2h optimization and PCM (DMF)||-1,2|S,0.,0.9645557579,1.299533
5457|S,0.,-0.9645557579,1.2995335457|S,0.,-0.9645557579,-1.2995335457|
S,0.,0.9645557579,-1.2995335457||Version=EM64W-G16RevB.01|State=2-AU|H
F=-1592.4716101|S2=0.755011|S2-1=0.|S2A=0.750023|RMSD=8.509e-009|RMSF=
2.168e-005|Dipole=0.,0.,0.|Quadrupole=2.4286483,2.5159367,-4.944585,0.
,0.,0.|PG=D02H [SG(S4)]||@

UPBE0/D3/Def2-QZVP geometry optimization for $S_5^{\bullet-}$ in C_s.

1|1|UNPC-DESKTOP-3H3V0JT|FOpt|UPBE1PBE|def2QZVP|S5(1-,2)|HOME|10-May-2
023|0||# opt geom=connectivity def2qzvp empiricaldispersion=gd3 upbe1p
be||upbe1pbe/D3/def2-qzvp tent S5- radical anion in Cs symmetry gas ph
ase||-1,2|S,0.45414612,-0.58101407,1.59177237|S,-0.13222088,1.24690104
,2.04744117|S,0.45414612,-0.58101407,-1.59177237|S,-0.13222088,1.24690
104,-2.04744117|S,-0.64385048,-1.33177394,0.||Version=EM64W-G16RevB.01
|State=2-A"|HF=-1990.4924098|S2=0.763479|S2-1=0.|S2A=0.750094|RMSD=6.0
71e-009|RMSF=4.839e-006|Dipole=0.0701124,-1.0610119,0.|Quadrupole=5.38
85542,1.819489,-7.2080432,-0.1503546,0.,0.|PG=CS [SG(S1),X(S4)]||@

UPBE0/D3/Def2-QZVP geometry optimization for $S_5^{\bullet-}$ in C_s with PCM=DMF.

1|1|UNPC-TABLET|FOpt|UPBE1PBE|def2QZVP|S5(1-,2)|USER|23-Apr-2023|0||#
opt upbe1pbe/def2qzvp scrf=(solvent=n,n-dimethylformamide,pcm) geom=co
nnectivity empiricaldispersion=gd3||upbe1pbe/D3/def2-qzvp tent S5- rad
ical anion cs symmetry with PCM (DMF)||-1,2|S,0.2546682419,-0.69330793
44,1.5583928895|S,0.2565512722,1.2733222502,1.8068222326|S,0.254668241
9,-0.6933079344,-1.5583928895|S,0.2565512722,1.2733222502,-1.806822232
6|S,-1.0224390481,-1.1600286215,0.||Version=EM64W-G16RevB.01|State=2-A
"|HF=-1990.5672752|S2=0.759985|S2-1=0.|S2A=0.750064|RMSD=7.297e-009|RM
SF=5.155e-005|Dipole=-0.385059,-1.5464737,0.|Quadrupole=3.9057306,1.31
51089,-5.2208394,-0.9316932,0.,0.|PG=CS [SG(S1),X(S4)]||@

Table S6 Gaussian archive entries for $S_5^{\bullet-}$

UPBE0/D3/Def2-QZVP geometry optimization for $S_5^{\bullet-}$ in C_{2v}.

```
1|1|UNPC-DESKTOP-3H3V0JT|FOpt|UPBE1PBE|def2QZVP|S5(1-,2)|HOME|10-May-2
 023|0||# opt geom=connectivity def2qzvp empiricaldispersion=gd3 upbe1p
 be||upbe1pbe/D3/def2-qzvp flat S5- radical anion in C2v symmetry gas p
 hase||-1,2|S,0.,1.87816634,0.59131135|S,0.,2.0138601,-1.36186062|S,0.,
 -1.87816634,0.59131135|S,0.,-2.0138601,-1.36186062|S,0.,0.,1.54109853|
 |Version=EM64W-G16RevB.01|State=2-B1|HF=-1990.4660167|S2=0.754936|S2-1
 =0.|S2A=0.750022|RMSD=5.379e-009|RMSF=7.541e-006|Dipole=0.,0.,1.086555
 5|Quadrupole=5.6871214,-7.4591502,1.7720288,0.,0.,0.|PG=C02V [C2(S1),S
 GV(S4)]||@
```

UPBE0/D3/Def2-QZVP geometry optimization for $S_5^{\bullet-}$ in C_{2v} with PCM=DMF.

```
1|1|UNPC-DESKTOP-3H3V0JT|FOpt|UPBE1PBE|def2QZVP|S5(1-,2)|HOME|06-May-2
 023|0||# opt upbe1pbe/def2qzvp scrf=(solvent=n,n-dimethylformamide,pcm
 ) geom=connectivity empiricaldispersion=gd3||upbe1pbe/D3/def2-qzvp fla
 t S5- radical anion C2v symmetry with PCM (DMF)||-1,2|S,0.,1.850451829
 ,0.592465355|S,0.,1.94114221,-1.3734750163|S,0.,-1.850451829,0.5924653
 55|S,0.,-1.94114221,-1.3734750163|S,0.,0.,1.5620193126||Version=EM64W-
 G16RevB.01|State=2-B1|HF=-1990.5401103|S2=0.754974|S2-1=0.|S2A=0.75002
 3|RMSD=5.380e-009|RMSF=1.021e-004|Dipole=0.,0.,1.7785039|Quadrupole=4.
 9055876,-6.6621556,1.756568,0.,0.,0.|PG=C02V [C2(S1),SGV(S4)]||@
```

UPBE0/D3/Def2-QZVP geometry optimization for $S_5^{\bullet-}$ in C_2.

```
1|1|UNPC-DESKTOP-3H3V0JT|FOpt|UPBE1PBE|def2QZVP|S5(1-,2)|HOME|10-May-2
 023|0||# opt geom=connectivity def2qzvp empiricaldispersion=gd3 upbe1p
 be||upbe1pbe/D3/def2-qzvp optimizaton of open chain S5- radical anion
 with C2symmetry||-1,2|S,0.7245687301,1.4102007199,-0.28742996|S,-0.332
 28954,3.07499103,-0.23942193|S,-0.7245687301,-1.4102007205,-0.28742995
 74|S,0.33228954,-3.0749910304,-0.2394219244|S,0.,0.000000001,1.0537037
 8||Version=EM64W-G16RevB.01|State=2-B|HF=-1990.4923997|S2=0.761784|S2-
 1=0.|S2A=0.750077|RMSD=8.071e-009|RMSF=1.949e-005|Dipole=0.,0.,0.26487
 17|Quadrupole=9.3502923,-18.0516148,8.7013225,1.7758433,0.,0.|PG=C02 [
 C2(S1),X(S4)]||@
```

UPBE0/D3/Def2-QZVP geometry optimization for $S_5^{\bullet-}$ in C_2 with PCM=DMF.

```
1|1|UNPC-DESKTOP-3H3V0JT|FOpt|UPBE1PBE|def2QZVP|S5(1-,2)|HOME|23-Apr-2
 023|0||# opt upbe1pbe/def2qzvp scrf=(solvent=n,n-dimethylformamide,pcm
 ) geom=connectivity empiricaldispersion=gd3||upbe1pbe/D3/def2-qzvp spi
 nner S5- radical anion C2 symmetry with PCM (DMF)||-1,2|S,0.0069879517
 ,1.5664665338,-0.2633136659|S,-1.732825209,2.500438358,-0.2829099024|S
 ,-0.0069879517,-1.5664665338,-0.2633136659|S,1.732825209,-2.500438358,
 -0.2829099024|S,0.,0.,1.0924471366||Version=EM64W-G16RevB.01|State=2-B
 |HF=-1990.5624258|S2=0.760729|S2-1=0.|S2A=0.750068|RMSD=7.435e-009|RMS
 F=1.588e-005|Dipole=0.,0.,0.413872|Quadrupole=1.3267481,-9.8389511,8.5
 122031,12.5219607,0.,0.|PG=C02 [C2(S1),X(S4)]||@
```

Table S7 Gaussian archive entries for $S_6^{\bullet-}$

UPBE0/D3/Def2-QZVP geometry optimization for $S_6^{\bullet-}$ in C_{2h}.

```
1|1|UNPC-DESKTOP-3H3V0JT|FOpt|UPBE1PBE|def2QZVP|S6(1-,2)|HOME|10-May-2
 023|0||# opt geom=connectivity def2qzvp empiricaldispersion=gd3 upbe1p
 be||upbe1pbe/D3/def2-qzvp optimization of gas phase cyclic S6- radical
  anion in C2h||-1,2|S,-0.40808165,1.97474038,0.|S,0.40808165,1.1056278
 8,1.62771064|S,-0.40808165,-1.10562788,1.62771064|S,0.40808165,-1.9747
 4038,0.|S,-0.40808165,-1.10562788,-1.62771064|S,0.40808165,1.10562788,
 -1.62771064||Version=EM64W-G16RevB.01|State=2-BG|HF=-2388.5786651|S2=0
 .759537|S2-1=0.|S2A=0.750053|RMSD=5.585e-009|RMSF=8.650e-005|Dipole=0.
 ,0.,0.|Quadrupole=4.3893865,-2.4616805,-1.927706,-1.2515217,0.,0.|PG=C
 02H [SGH(S2),X(S4)]||@
```

UPBE0/D3/Def2-QZVP geometry optimization for $S_6^{\bullet-}$ in C_{2h} with PCM=DMF.

```
1|1|UNPC-DESKTOP-3H3V0JT|FOpt|UPBE1PBE|def2QZVP|S6(1-,2)|HOME|22-Apr-2
 023|0||# opt upbe1pbe/def2qzvp scrf=(solvent=n,n-dimethylformamide,pcm
 ) geom=connectivity empiricaldispersion=gd3||upbe1pbe/D3/def2-qzvp opt
 imization of cyclic S6- radical anion in C2h - with PCM (DMF)||-1,2|S,
 -0.4401993833,1.9692563948,0.0000000007|S,0.3897860485,1.1120556115,1.
 6265897443|S,-0.3897860485,-1.1120556127,1.6265897435|S,0.4401993833,-
 1.9692563948,-0.0000000007|S,-0.3897860485,-1.1120556115,-1.6265897443
 |S,0.3897860485,1.1120556127,-1.6265897435||Version=EM64W-G16RevB.01|S
 tate=2-BG|HF=-2388.6484224|S2=0.759734|S2-1=0.|S2A=0.750055|RMSD=4.477
 e-009|RMSF=3.386e-005|Dipole=0.,0.,0.|Quadrupole=3.4959574,-1.8267669,
 -1.6691905,-1.3647346,0.,0.|PG=C02H [SGH(S2),X(S4)]||@
```

UPBE0/D3/Def2-QZVP geometry optimization for $S_6^{\bullet-}$ in C_2 ring.

```
1|1|UNPC-DESKTOP-3H3V0JT|FOpt|UPBE1PBE|def2QZVP|S6(1-,2)|HOME|10-May-2
 023|0||# opt geom=connectivity def2qzvp empiricaldispersion=gd3 upbe1p
 be||uPBE0/D3/def2-qzvp optimization of S6- radical anion in C2 symmetr
 y||-1,2|S,0.42706054,1.96343327,-0.04107525|S,-0.44151753,0.93080185,-
 1.61255511|S,0.44151753,-0.93080185,-1.61255511|S,-0.42706054,-1.96343
 327,-0.04107525|S,0.44151753,-1.34072018,1.65363036|S,-0.44151753,1.34
 072018,1.65363036||Version=EM64W-G16RevB.01|State=2-B|HF=-2388.5872402
 |S2=0.756255|S2-1=0.|S2A=0.750034|RMSD=6.956e-009|RMSF=4.445e-005|Dipo
 le=0.,0.,-0.9462519|Quadrupole=4.7636203,-3.1957271,-1.5678933,1.24375
 9,0.,0.|PG=C02 [X(S6)]||@
```

UPBE0/D3/Def2-QZVP geometry optimization for $S_6^{\bullet-}$ in C_2 ring with PCM=DMF.

```
1|1|UNPC-DESKTOP-3H3V0JT|FOpt|UPBE1PBE|def2QZVP|S6(1-,2)|HOME|21-Apr-2
 023|0||# opt upbe1pbe/def2qzvp geom=connectivity empiricaldispersion=g
 d3 scrf=(solvent=n,n-dimethylformamide,pcm)||upbe1be/D3/def2-qzvp opti
 mization of cyclic S6- radical anion in C2 symmetry with PCM (DMF)||-1
 ,2|S,0.48305023,1.95030929,-0.04717171|S,-0.41937006,0.93762192,-1.606
 85922|S,0.41937006,-0.9376219239,-1.6068592177|S,-0.48305023,-1.950309
 2901,-0.0471717053|S,0.40580549,-1.35258593,1.65403093|S,-0.40580549,1
 .352585934,1.6540309268||Version=EM64W-G16RevB.01|State=2-B|HF=-2388.6
 587467|S2=0.756109|S2-1=0.|S2A=0.750032|RMSD=5.805e-009|RMSF=5.518e-00
 5|Dipole=0.,0.,-1.5257678|Quadrupole=4.0294789,-2.7276813,-1.3017976,1
 .2649862,0.,0.|PG=C02 [X(S6)]||@
```

Table S8 Gaussian archive entries for $S_6^{\bullet-}$ and $S_7^{\bullet-}$

UPBE0/D3/Def2-QZVP geometry optimization for $S_6^{\bullet-}$ in C_2 open chain


```
1|1|UNPC-DESKTOP-3H3V0JT|FOpt|UPBE1PBE|def2QZVP|S6(1-,2)|HOME|10-May-2
 023|0||# opt geom=connectivity def2qzvp empiricaldispersion=gd3 upbe1p
 be||upbe1pbe/D3/def2-qzvp optimization of open chain S6- radical anion
  in C2 symmetry||-1,2|S,0.9467265878,-2.0032992483,-0.6659373803|S,0.9
 479433026,-0.4994063027,0.6924926957|S,-0.9479433026,0.4994063027,0.69
 24926957|S,-0.9467265878,2.0032992483,-0.6659373803|S,-0.0291086276,3.
 6267766067,-0.0265553155|S,0.0291086276,-3.6267766067,-0.0265553155||V
 ersion=EM64W-G16RevB.01|State=2-A|HF=-2388.5876029|S2=0.778711|S2-1=0.
 |S2A=0.750239|RMSD=8.585e-009|RMSF=1.821e-005|Dipole=0.,0.,-0.0338096|
 Quadrupole=11.6769158,-23.3443069,11.6673911,2.0280708,0.,0.|PG=C02 [X
 (S6)]||@
```


UPBE0/D3/Def2-QZVP geometry optimization for $S_6^{\bullet-}$ in C_2 open chain with PCM=DMF.


```
1|1|UNPC-DESKTOP-3H3V0JT|FOpt|UPBE1PBE|def2QZVP|S6(1-,2)|HOME|01-Apr-2
 023|0||# opt upbe1pbe/def2qzvp geom=connectivity g09defaults scrf=(sol
 vent=n,n-dimethylformamide,pcm)||uPBE0/def2-qzvp cyclic S6- radical an
 ion in twisted C2 with PCM (DMF)||-1,2|S,-0.6606850602,2.0730707424,-0
 .6672204552|S,-0.8726876585,0.6127725982,0.719054788|S,0.8726876585,-0
 .6127725982,0.719054788|S,0.6606850602,-2.0730707424,-0.6672204552|S,-
 0.5444478326,-3.5109177147,-0.0518343471|S,0.5444478326,3.5109177147,-
 0.0518343471||Version=EM64W-G16RevB.01|State=2-A|HF=-2388.6505322|S2=0
 .774795|S2-1=0.|S2A=0.750198|RMSD=3.862e-009|RMSF=5.724e-005|Dipole=0.
 ,0.,-0.0555616|Quadrupole=11.4140804,-22.8663018,11.4522214,-4.374502,
 0.,0.|PG=C02 [X(S6)]||@
```


UPBE0/D3/Def2-QZVP geometry optimization for $S_7^{\bullet-}$ in C_s chair


```
1|1|UNPC-DESKTOP-3H3V0JT|FOpt|UPBE1PBE|def2QZVP|S7(1-,2)|HOME|10-May-2
 023|0||# opt geom=connectivity def2qzvp empiricaldispersion=gd3 upbe1p
 be||upbe1pbe/D3/def2-qzvp optimization of chair S7 radical anion in Cs
  symmetry||-1,2|S,1.46970125,-1.66045214,0.|S,0.33393117,-1.3488371,1.
 67937066|S,0.33393117,0.70306527,2.03407462|S,-1.40271297,1.4759979,1.
 47305592|S,-1.40271297,1.4759979,-1.47305592|S,0.33393117,0.70306527,-
 2.03407462|S,0.33393117,-1.3488371,-1.67937066||Version=EM64W-G16RevB.
 01|State=2-A"|HF=-2786.6861315|S2=0.757255|S2-1=0.|S2A=0.750042|RMSD=7
 .439e-009|RMSF=3.086e-005|Dipole=0.8267154,-0.9135229,0.|Quadrupole=3.
 5194833,0.1833369,-3.7028202,5.0725199,0.,0.|PG=CS [SG(S1),X(S6)]||@
```


UPBE0/D3/Def2-QZVP geometry optimization for $S_7^{\bullet-}$ in C_s chair with PCM=DMF.


```
1|1|UNPC-DESKTOP-3H3V0JT|FOpt|UPBE1PBE|def2QZVP|S7(1-,2)|HOME|20-Apr-2
 023|0||# opt upbe1pbe/def2qzvp scrf=(solvent=n,n-dimethylformamide,pcm
 ) geom=connectivity empiricaldispersion=gd3||upbe1pbe/D3/def2-qzvp opt
 imization of S7 radical anion in Cs symmetry in DMF||-1,2|S,0.25500168
 ,-2.19722419,0.|S,-0.50135626,-1.28991066,1.67348101|S,0.68481507,0.37
 777353,2.02330872|S,-0.31095965,2.01074923,1.47216165|S,-0.31095965,2.
 01074923,-1.47216165|S,0.68481507,0.37777353,-2.02330872|S,-0.50135626
 ,-1.28991066,-1.67348101||Version=EM64W-G16RevB.01|State=2-A"|HF=-2786
 .7547526|S2=0.756794|S2-1=0.|S2A=0.750037|RMSD=7.692e-009|RMSF=3.197e-
 005|Dipole=0.2341963,-2.0235687,0.|Quadrupole=6.4539197,-3.3721685,-3.
 0817512,0.4429869,0.,0.|PG=CS [SG(S1),X(S6)]||@
```

Table S9 Gaussian archive entries for $S_7^{\bullet-}$

UPBE0/D3/Def2-QZVP geometry optimization for $S_7^{\bullet-}$ in C_s boat

```
1|1|UNPC-DESKTOP-3H3V0JT|FOpt|UPBE1PBE|def2QZVP|S7(1-,2)|HOME|10-May-2
 023|0||# opt geom=connectivity def2qzvp empiricaldispersion=gd3 upbe1p
 be||upbe1pbe/D3/def2-qzvp optimization of boat S7 radical anion in Cs
 symmetry||-1,2|S,-0.95585623,1.6001999,1.59173732|S,-0.95585623,-0.351
 3521,1.79284459|S,1.00671168,-1.11444401,1.67279653|S,1.81000156,-0.26
 880758,0.|S,1.00671168,-1.11444401,-1.67279653|S,-0.95585623,-0.351352
 1,-1.79284459|S,-0.95585623,1.6001999,-1.59173732||Version=EM64W-G16Re
 vB.01|State=2-A"|HF=-2786.6840146|S2=0.758198|S2-1=0.|S2A=0.75005|RMSD
 =5.644e-009|RMSF=6.016e-005|Dipole=0.6366116,-0.954054,0.|Quadrupole=2
 .6613055,1.7873549,-4.4486604,4.4023355,0.,0.|PG=CS [SG(S1),X(S6)]||@
```

UPBE0/D3/Def2-QZVP geometry optimization for $S_7^{\bullet-}$ in C_s boat with PCM=DMF.

```
1|1|UNPC-DESKTOP-3H3V0JT|FOpt|UPBE1PBE|def2QZVP|S7(1-,2)|HOME|10-May-2
 023|0||# opt scrf=(solvent=n,n-dimethylformamide,pcm) geom=connectivit
 y def2qzvp empiricaldispersion=gd3 upbe1pbe||upbe1pbe/D3/def2-qzvp opt
 imization of boat S7 radical anion in Cs symmetry with PCM=DMF||-1,2|S
 ,-0.95503092,1.5995051,1.61434562|S,-0.95503092,-0.36279528,1.76982164
 |S,1.00319201,-1.10966278,1.66292587|S,1.81373966,-0.25409407,0.|S,1.0
 0319201,-1.10966278,-1.66292587|S,-0.95503092,-0.36279528,-1.76982164|
 S,-0.95503092,1.5995051,-1.61434562||Version=EM64W-G16RevB.01|State=2-
 A"|HF=-2786.753419|S2=0.757521|S2-1=0.|S2A=0.750044|RMSD=3.669e-009|RM
 SF=3.736e-005|Dipole=1.0683525,-1.6379131,0.|Quadrupole=2.9260862,1.07
 36794,-3.9997656,4.2952612,0.,0.|PG=CS [SG(S1),X(S6)]||@
```

UPBE0/D3/Def2-QZVP geometry optimization for $S_7^{\bullet-}$ in C_2 open chain

```
1|1|UNPC-DESKTOP-3H3V0JT|FOpt|UPBE1PBE|def2QZVP|S7(1-,2)|HOME|10-May-2
 023|0||# opt geom=connectivity def2qzvp empiricaldispersion=gd3 upbe1p
 be||upbe1pbe/D3/def2-qzvp optimization of S7 radical anion chain in C2
 ||-1,2|S,0.,0.,1.4828095838|S,1.0735532039,1.3247897898,0.314706902|S,
 0.0007590076,3.0834638651,0.2994094271|S,-1.0427650746,3.3148755646,-1
 .3555211261|S,1.0427650746,-3.3148755646,-1.3555211261|S,-0.0007590076
 ,-3.0834638651,0.2994094271|S,-1.0735532039,-1.3247897898,0.314706902|
 |Version=EM64W-G16RevB.01|State=2-B|HF=-2786.6760501|S2=0.773192|S2-1=
 0.|S2A=0.750177|RMSD=7.213e-009|RMSF=3.946e-006|Dipole=0.,0.,1.1207343
 |Quadrupole=10.7413595,-19.6959972,8.9546377,7.565309,0.,0.|PG=C02 [C2
 (S1),X(S6)]||@
```

UPBE0/D3/Def2-QZVP geometry optimization for $S_7^{\bullet-}$ in C_s open chain with PCM=DMF.

```
1|1|UNPC-DESKTOP-3H3V0JT|FOpt|UPBE1PBE|def2QZVP|S7(1-,2)|HOME|11-May-2
 023|0||# opt scrf=(solvent=n,n-dimethylformamide,pcm) geom=connectivit
 y def2qzvp empiricaldispersion=gd3 upbe1pbe||upbe1pbe/D3/def2-qzvp opt
 imization of S7 radical anion chain in C2 with DMF||-1,2|S,0.,0.,1.730
 1342683|S,1.1333043298,1.2433855825,0.544052757|S,-0.0182443923,2.9138
 50888,0.2022747667|S,-0.8070941892,2.8557481339,-1.6113946576|S,0.8070
 941892,-2.8557481339,-1.6113946576|S,0.0182443923,-2.913850888,0.20227
 47667|S,-1.1333043298,-1.2433855825,0.544052757||Version=EM64W-G16RevB
 .01|State=2-B|HF=-2786.7402724|S2=0.764572|S2-1=0.|S2A=0.750099|RMSD=9
 .798e-009|RMSF=1.225e-005|Dipole=0.,0.,2.0204201|Quadrupole=9.6068325,
 -14.3926378,4.7858053,6.2046684,0.,0.|PG=C02 [C2(S1),X(S6)]||@
```

Table S10 Gaussian archive entries for $S_8^{\bullet-}$

UPBE0/D3/Def2-QZVP geometry optimization for $S_8^{\bullet-}$ in C_{2v}

```
1|1|UNPC-DESKTOP-3H3V0JT|FOpt|UPBE1PBE|def2QZVP|S8(1-,2)|HOME|10-May-2
 023|0||# opt geom=connectivity def2qzvp empiricaldispersion=gd3 upbe1p
 be||upbe1pbe/D3/def2-qzvp optimization of gas phase S8 radical anion i
 n C2v symmetry||-1,2|S,0.,2.17511071,0.52226648|S,1.82831761,1.6591507
 7,-0.45036909|S,2.666015,0.,0.37847169|S,1.82831761,-1.65915077,-0.450
 36909|S,0.,-2.17511071,0.52226648|S,-1.82831761,-1.65915077,-0.4503690
 9|S,-2.666015,0.,0.37847169|S,-1.82831761,1.65915077,-0.45036909||Vers
 ion=EM64W-G16RevB.01|State=2-B2|HF=-3184.774715|S2=0.762139|S2-1=0.|S2
 A=0.75009|RMSD=4.009e-009|RMSF=2.125e-005|Dipole=0.,0.,-0.0410218|Quad
 rupole=-4.0938364,-3.6646706,7.758507,0.,0.,0.|PG=C02V [SGV(S2),SGV'(S
 2),X(S4)]||@
```

UPBE0/D3/Def2-QZVP geometry optimization for $S_8^{\bullet-}$ in C_{2v} with PCM=DMF.

```
1|1|UNPC-DESKTOP-3H3V0JT|FOpt|UPBE1PBE|def2QZVP|S8(1-,2)|HOME|11-May-2
 023|0||# opt scrf=(solvent=n,n-dimethylformamide,pcm) geom=connectivit
 y def2qzvp empiricaldispersion=gd3 upbe1pbe||upbe1pbe/D3/def2-qzvp opt
 imization of S8 radical anion in c2v symmetry with PCM (DMF)||-1,2|S,0
 .,2.1681063193,0.5179652322|S,1.8353341949,1.6559622976,-0.4512567532|
 S,2.6680555503,0.,0.3845482742|S,1.8353341949,-1.6559622976,-0.4512567
 532|S,0.,-2.1681063193,0.5179652322|S,-1.8353341949,-1.6559622976,-0.4
 512567532|S,-2.6680555503,0.,0.3845482742|S,-1.8353341949,1.6559622976
 ,-0.4512567532||Version=EM64W-G16RevB.01|State=2-B2|HF=-3184.8375592|S
 2=0.762484|S2-1=0.|S2A=0.750094|RMSD=6.539e-009|RMSF=6.091e-006|Dipole
 =0.,0.,-0.095888|Quadrupole=-2.3003775,-4.0452195,6.345597,0.,0.,0.|PG
 =C02V [SGV(S2),SGV'(S2),X(S4)]||@
```

UPBE0/D3/Def2-QZVP geometry optimization for $S_8^{\bullet-}$ in C_2 ring

```
1|1|UNPC-TABLET|FOpt|UPBE1PBE|def2QZVP|S8(1-,2)|USER|20-Apr-2023|0||#
 opt upbe1pbe/def2qzvp geom=connectivity empiricaldispersion=gd3||upbe1
 pbe/D3/def2-qzvp gas phase optimization of S8 radical anion in C2 symm
 etry||-1,2|S,-0.5359532801,0.8764132204,-2.1372579943|S,0.3880323097,2
 .2983839459,-0.9678536055|S,-0.4747624089,2.3663146664,0.8999317636|S,
 0.5573430205,1.2685582006,2.2051798363|S,-0.5573430205,-1.2685581948,2
 .2051798396|S,0.4747624089,-2.366314664,0.8999317698|S,-0.3880323097,-
 2.2983839484,-0.9678535994|S,0.5359532801,-0.876413226,-2.137257992||V
 ersion=EM64W-G16RevB.01|State=2-B|HF=-3184.7825593|S2=0.756626|S2-1=0.
 |S2A=0.750037|RMSD=4.752e-009|RMSF=2.377e-005|Dipole=0.,0.,-1.3198095|
 Quadrupole=8.2263464,-3.69407,-4.5322764,-1.3861414,0.,0.|PG=C02 [X(S8
 )]||@
```

UPBE0/D3/Def2-QZVP geometry optimization for $S_8^{\bullet-}$ in C_2 ring with PCM=DMF.

```
1|1|UNPC-DESKTOP-3H3V0JT|FOpt|UPBE1PBE|def2QZVP|S8(1-,2)|HOME|20-Apr-2
 023|0||# opt upbe1pbe/def2qzvp scrf=(solvent=n,n-dimethylformamide,pcm
 ) geom=connectivity empiricaldispersion=gd3||upbe1pbe/D3/def2-qzvp opt
 imization of S8 radical anion in C2 symmetry with PCM||-1,2|S,-0.54567
 18526,0.8693272676,-2.1216075818|S,0.3878440629,2.2928828403,-0.970218
 443|S,-0.4780284692,2.3712879007,0.8925535721|S,0.5658523327,1.2706694
 238,2.1992724527|S,-0.5658523327,-1.2706694217,2.1992724539|S,0.478028
 4692,-2.3712878998,0.8925535744|S,-0.3878440629,-2.2928828412,-0.97021
 84407|S,0.5456718526,-0.8693272697,-2.121607581||Version=EM64W-G16RevB
 .01|State=2-B|HF=-3184.8484595|S2=0.75634|S2-1=0.|S2A=0.750034|RMSD=2.
 904e-009|RMSF=2.354e-005|Dipole=0.,0.,-2.2252462|Quadrupole=7.0241138,
 -2.2599708,-4.764143,-1.8504312,0.,0.|PG=C02 [X(S8)]||@
```

Table S11 Gaussian archive entries for $S_8^{\bullet-}$

UPBE0/D3/Def2-QZVP geometry optimization for $S_8^{\bullet-}$ in C_2 chain

```
1|1|UNPC-DESKTOP-3H3V0JT|FOpt|UPBE1PBE|def2QZVP|S8(1-,2)|HOME|11-May-2
 023|0||# opt geom=connectivity def2qzvp empiricaldispersion=gd3 upbe1p
 be||upbe1pbe/D3/def2-qzvp optimization of S8- radical anion with C2 ch
 ain symmetry||-1,2|S,-0.9311516262,0.4100022087,-0.3985660813|S,-0.392
 3400231,3.6492238137,-0.2011488205|S,1.5685924541,3.8131580309,-0.3203
 13958|S,-0.931093955,2.0079198195,0.9200288597|S,0.9311516262,-0.41000
 22087,-0.3985660813|S,0.931093955,-2.0079198195,0.9200288597|S,0.39234
 00231,-3.6492238137,-0.2011488205|S,-1.5685924541,-3.8131580309,-0.320
 313958||Version=EM64W-G16RevB.01|State=2-A|HF=-3184.7702695|S2=0.77132
 4|S2-1=0.|S2A=0.750161|RMSD=8.282e-009|RMSF=6.457e-006|Dipole=0.,0.,0.
 2867827|Quadrupole=11.2955503,-27.1788969,15.8833466,-11.5876896,0.,0.
 |PG=C02 [X(S8)]||@
```

UPBE0/D3/Def2-QZVP geometry optimization for $S_8^{\bullet-}$ in C_{2v} chain with PCM=DMF.

```
1|1|UNPC-DESKTOP-3H3V0JT|FOpt|UPBE1PBE|def2QZVP|S8(1-,2)|HOME|11-May-2
 023|0||# opt scrf=(solvent=n,n-dimethylformamide,pcm) geom=connectivit
 y def2qzvp empiricaldispersion=gd3 upbe1pbe||upbe1pbe/D3/def2-qzvp S8-
  radical anion with C2 chain symmetry in DMF||-1,2|S,-0.938451103,0.39
 40221842,-0.380787261|S,-0.2933191159,3.5722012759,-0.199654267|S,1.67
 77239026,3.5134014924,-0.3579050967|S,-0.9418233466,1.9899266443,0.938
 3466245|S,0.938451103,-0.3940221842,-0.380787261|S,0.9418233466,-1.989
 9266443,0.9383466245|S,0.2933191159,-3.5722012759,-0.199654267|S,-1.67
 77239026,-3.5134014924,-0.3579050967||Version=EM64W-G16RevB.01|State=2
 -A|HF=-3184.8319582|S2=0.768243|S2-1=0.|S2A=0.750133|RMSD=4.580e-009|R
 MSF=1.076e-005|Dipole=0.,0.,0.4989251|Quadrupole=8.7442475,-22.952753,
 14.2085055,-14.8869579,0.,0.|PG=C02 [X(S8)]||@
```

Table S12 Frequency calculations on $S_4^{\bullet -}$ optimizations

UPBE0/D3/Def2-QZVP harmonic frequencies (cm^{-1}) and IR intensities (km/mol):

$S_4^{\bullet -}$ C_{2v} symmetry optimization

	1	2	3
	A2	A1	B2
Frequencies --	93.4793	99.2629	297.1073
IR Inten --	0.0000	0.2335	0.9587
	4	5	6
	A1	B2	A1
Frequencies --	372.2304	597.4382	629.4088
IR Inten --	5.3568	226.4845	11.2075

$S_4^{\bullet -}$ C_{2h} symmetry optimization

	1	2	3
	AU	BU	AG
Frequencies --	61.1152	116.6634	211.2402
IR Inten --	2.5946	4.0829	0.0000
	4	5	6
	AG	BU	AG
Frequencies --	491.5503	571.9509	601.5166
IR Inten --	0.0000	394.8908	0.0000

$S_4^{\bullet -}$ D_{2h} symmetry optimization

	1	2	3
	B2U	AU	AG
Frequencies --	-158.9028	186.1824	257.6270
IR Inten --	26.1194	0.0000	0.0000
	4	5	6
	B3G	B1U	AG
Frequencies --	297.1280	620.4993	690.2382
IR Inten --	0.0000	246.9307	0.0000

Table S13 Frequency calculations on $S_5^{\bullet-}$ optimizations

UPBE0/D3/Def2-QZVP harmonic frequencies (cm^{-1}) and IR intensities (km/mol):

$S_5^{\bullet-}$ Cs symmetry optimization

```
                      1                      2                      3
                      A'                     A"                     A'
Frequencies --     25.0874                79.4777               179.7227
IR Inten    --      0.0345                 0.8430                 1.0969
                      4                      5                      6
                      A"                     A'                     A"
Frequencies --    183.8229               253.4055               380.3830
IR Inten    --      3.0693                 0.5333                 5.2795
                      7                      8                      9
                      A'                     A"                     A'
Frequencies --    465.9370               565.8056               591.0893
IR Inten    --      9.3689               308.6005                28.0072
```

$S_5^{\bullet-}$ C_2 symmetry optimization

```
                      1                      2                      3
                      B                      A                      A
Frequencies --     45.5749                46.8312               142.8699
IR Inten    --      6.3423                 1.6670                 0.1016
                      4                      5                      6
                      A                      B                      B
Frequencies --    167.5055               231.4492               381.4829
IR Inten    --      0.4447                16.5141                 2.2209
                      7                      8                      9
                      A                      B                      A
Frequencies --    475.2427               574.6505               598.9152
IR Inten    --      3.3375               608.9529                 3.9688
```

$S_5^{\bullet-}$ C_{2v} symmetry optimization

```
                      1                      2                      3
                     B1                     A2                     A1
Frequencies --   -179.6909              -101.6574                70.4015
IR Inten    --      0.5337                 0.0000                 0.0409
                      4                      5                      6
                     B2                     A1                     A1
Frequencies --    130.6248               321.6024               350.9349
IR Inten    --      4.2669                 0.8261                15.1348
                      7                      8                      9
                     B2                     B2                     A1
Frequencies --    380.5537               573.6585               628.8216
IR Inten    --     65.2056               306.3383                52.9086
```

Table S14 Frequency calculations on $S_6^{\bullet-}$ optimizations

UPBE0/D3/Def2-QZVP harmonic frequencies (cm^{-1}) and IR intensities (km/mol):

$S_6^{\bullet-}$ C_{2h} symmetry optimization

	1	2	3
	AU	BG	BU
Frequencies --	-274.2818	61.7422	134.0931
IR Inten --	163.0819	0.0000	1.0085
	**4**	**5**	**6**
	AU	AG	AG
Frequencies --	136.1077	175.9355	237.9972
IR Inten --	0.4085	0.0000	0.0000
	**7**	**8**	**9**
	AG	BU	BU
Frequencies --	267.0270	277.8538	492.5954
IR Inten --	0.0000	2.1517	23.7686
	**10**	**11**	**12**
	AU	BG	AG
Frequencies --	501.9027	504.4655	516.7799
IR Inten --	38.7977	0.0000	0.0000

$S_6^{\bullet-}$ C_2 (chair) symmetry optimization

	1	2	3
	A	B	B
Frequencies --	94.7215	96.5858	156.4278
IR Inten --	0.2605	2.1264	4.8446
	**4**	**5**	**6**
	A	A	A
Frequencies --	161.4525	193.2940	237.5156
IR Inten --	1.6295	1.1399	1.9310
	**7**	**8**	**9**
	B	A	B
Frequencies --	277.1331	412.7085	434.5716
IR Inten --	0.4366	0.7120	0.8233
	**10**	**11**	**12**
	A	B	A
Frequencies --	468.8924	524.0344	535.0498
IR Inten --	13.3616	40.6943	24.9367

$S_6^{\bullet-}$ C_2 (chain) symmetry optimization

	1	2	3
	A	B	A
Frequencies --	33.2114	40.8637	78.7024
IR Inten --	1.4966	3.3952	0.0540
	**4**	**5**	**6**
	A	B	B
Frequencies --	110.7337	193.9436	226.2836
IR Inten --	0.0091	14.1353	0.2443
	**7**	**8**	**9**
	A	A	A
Frequencies --	265.5562	305.3823	464.6657
IR Inten --	2.0085	1.5687	0.6256
	**10**	**11**	**12**
	B	B	A
Frequencies --	481.8776	574.6453	594.1989
IR Inten --	75.9763	772.2967	0.6326

Table S15 Frequency calculations on $S_7^{\bullet-}$ optimizations

UPBE0/D3/Def2-QZVP harmonic frequencies (cm^{-1}) and IR intensities (km/mol):

$S_7^{\bullet-}$ C_s (chair) symmetry optimization

	1	2	3
	A"	A'	A"
Frequencies --	55.0680	67.3641	80.0563
IR Inten --	0.1628	0.1122	0.2565
	4	5	6
	A'	A"	A'
Frequencies --	147.6834	165.8997	170.3570
IR Inten --	1.1496	6.6350	1.5937
	7	8	9
	A"	A'	A'
Frequencies --	215.1894	218.4226	252.0467
IR Inten --	1.1153	4.5561	1.0412
	10	11	12
	A"	A'	A"
Frequencies --	400.4765	412.2816	466.8077
IR Inten --	2.5945	3.3965	0.8229
	13	14	15
	A'	A"	A'
Frequencies --	480.0905	555.7341	561.8168
IR Inten --	5.5876	42.3251	43.5151

$S_7^{\bullet-}$ C_s (boat) symmetry optimization

	1	2	3
	A"	A"	A'
Frequencies --	-42.9779	73.8807	83.3369
IR Inten --	5.2028	6.0109	2.7333
	4	5	6
	A'	A"	A'
Frequencies --	101.9495	137.4274	141.7754
IR Inten --	5.1021	2.0189	0.3179
	7	8	9
	A'	A"	A'
Frequencies --	238.2868	243.4486	303.7557
IR Inten --	5.7008	1.2116	5.1609
	10	11	12
	A'	A"	A"
Frequencies --	406.0886	406.3419	456.1555
IR Inten --	27.3847	22.2005	28.0512
	13	14	15
	A'	A"	A'
Frequencies --	493.6848	548.5758	585.2342
IR Inten --	3.7015	443.5795	92.8731

Table S16 Frequency calculations on $S_7^{•-}$ and $S_8^{•-}$ optimizations

UPBE0/D3/Def2-QZVP harmonic frequencies (cm^{-1}) and IR intensities (km/mol):

$S_7^{•-}$ C_2 (chain) symmetry optimization

	1	2	3
	A	A	B
Frequencies --	22.8317	39.9410	45.9331
IR Inten --	1.1174	0.1441	0.2275
	4	5	6
	B	A	A
Frequencies --	56.7017	135.3776	171.4867
IR Inten --	5.2890	0.7966	1.0865
	7	8	9
	B	B	A
Frequencies --	200.1927	213.4079	239.9480
IR Inten --	44.8596	8.2971	1.2740
	10	11	12
	B	A	B
Frequencies --	392.1918	426.2889	466.2292
IR Inten --	72.2840	0.0819	50.9643
	13	14	15
	A	B	A
Frequencies --	471.3154	572.7738	590.5036
IR Inten --	1.0272	776.7433	29.0675

$S_8^{•-}$ C_{2v} (crown) symmetry optimization

	1	2	3
	A"	A"	A'
Frequencies --	-215.2462	51.8209	67.1894
IR Inten --	64.3470	0.2155	2.1094
	4	5	6
	A"	A'	A'
Frequencies --	113.0806	117.3600	158.5904
IR Inten --	0.0646	2.0549	12.0910
	7	8	9
	A"	A'	A'
Frequencies --	181.9323	207.1759	213.5920
IR Inten --	9.2254	0.9629	0.2477
	10	11	12
	A'	A"	A'
Frequencies --	233.3355	241.3447	329.3562
IR Inten --	1.5167	0.0205	11.4395
	13	14	15
	A"	A'	A'
Frequencies --	394.5500	441.2564	462.7533
IR Inten --	0.4552	3.0782	14.0967
	16	17	18
	A"	A"	A'
Frequencies --	463.9082	499.4670	505.1848
IR Inten --	1.7017	4.3092	11.1877

Table S17 Frequency calculations on $S_8^{\bullet-}$ optimizations

UPBE0/D3/Def2-QZVP harmonic frequencies (cm^{-1}) and IR intensities (km/mol):

$S_8^{\bullet-}$ C_2 (crown) symmetry optimization

	1	2	3
	A	B	B
Frequencies --	49.4216	57.0518	59.3085
IR Inten --	0.8422	1.0937	1.5085
	**4**	**5**	**6**
	A	A	B
Frequencies --	80.9170	154.6850	171.8857
IR Inten --	0.0549	6.1181	10.9404
	**7**	**8**	**9**
	A	A	B
Frequencies --	178.5849	203.5001	209.6522
IR Inten --	0.7127	8.5594	7.5327
	**10**	**11**	**12**
	B	A	A
Frequencies --	235.1542	242.9119	429.0432
IR Inten --	6.1976	0.4274	0.4327
	**13**	**14**	**15**
	B	A	B
Frequencies --	436.4123	469.0929	473.0558
IR Inten --	5.6644	0.0048	2.7528
	**16**	**17**	**18**
	A	B	A
Frequencies --	478.4379	528.0651	534.0523
IR Inten --	7.5720	60.7796	82.5429

$S_8^{\bullet-}$ C_2 (chain) symmetry optimization

	1	2	3
	A	B	A
Frequencies --	21.7359	41.3018	42.3835
IR Inten --	3.2937	1.3751	0.1205
	**4**	**5**	**6**
	A	B	A
Frequencies --	58.5326	64.2764	136.1222
IR Inten --	0.5991	16.7639	0.0625
	**7**	**8**	**9**
	B	A	B
Frequencies --	156.0179	202.7239	213.0387
IR Inten --	177.4056	0.2190	77.8752
	**10**	**11**	**12**
	A	B	A
Frequencies --	258.4979	273.7691	398.3972
IR Inten --	2.6225	51.3383	0.3340
	**13**	**14**	**15**
	B	A	B
Frequencies --	413.7622	471.5814	477.0589
IR Inten --	456.7368	7.7087	93.6209
	**16**	**17**	**18**
	A	A	B
Frequencies --	490.6968	577.8644	845.1786
IR Inten --	1.0823	8.0888	142811.3466
